# Injury characteristics and pre- to post-injury market-value change in professional football: a retrospective cohort study of 12,948 injury episodes

**DOI:** 10.3389/fpubh.2026.1966215

**Published:** 2026-10-01

**Authors:** Celal Gençoğlu, Hikmet Gümüş, Zarife Pancar, Yücel Makaracı

**Affiliations:** 1 Physical Education and Sports Department, Necat Hepkon Faculty of Sport Sciences, Dokuz Eylül University, İzmir, Türkiye; 2 Department of Coach Education, Necat Hepkon Faculty of Sport Sciences, Dokuz Eylül University, İzmir, Türkiye; 3 Department of Physical Education and Sports, Faculty of Sports Science, Gaziantep University, Gaziantep, Türkiye; 4 Department of Coaching Education, Faculty of Sports Sciences, Karamanoğlu Mehmetbey University, Karaman, Türkiye

**Keywords:** football analytics, football injuries, injury burden, player market value, transfer market

## Abstract

**Introduction:**

Player market value is a key indicator of a professional football player’s sporting and economic worth, yet how injury characteristics relate to the change in valuation after an injury remains poorly understood.

**Methods:**

This retrospective cohort study examined the association between injury characteristics and the pre- to post-injury change in market value using Transfermarkt data. The cohort comprised the complete injury histories (July 2008 to August 2024) of the 2,624 players listed in the 2024 squads of the English, Spanish, Italian, German and French top divisions; 12,948 injury episodes from 2,061 players had a valuation in effect 120 days before the injury start and a valuation update after the 120 days following the injury end, and the outcome was the percentage change between these two valuations. Kruskal–Wallis tests with effect sizes, Dunn pairwise comparisons and linear regression models with player-clustered standard errors were used; age interactions were tested with joint Wald tests, and an exploratory matched-control comparison assessed differences in log-value change between injured players and injury-free player-periods matched on league, position group, age and baseline market value.

**Results:**

The median change was 0.0% (interquartile range −25.0 to 66.7) and the mean 64.7%. Differences between injury categories and between duration groups were statistically significant but negligible in size (η^2^H ≤ 0.0025), whereas age accounted for a large share of the variation (η^2^H = 0.222). In the fully adjusted model each year of age was associated with −18.2 percentage points, and injuries of 31–90 and >90 days with −23.6 and −26.4 percentage points lower change than injuries of 1–14 days; injury category was not associated with the change, and no age-by-category or age-by-duration interaction was found (*p* = 0.940 and *p* = 0.125). In the matched-control analysis injured players showed −4.4% lower value growth than controls, reaching −26.9% for injuries longer than 90 days.

**Discussion:**

Injury duration, rather than injury category, is associated with a modest injury-related shortfall in market value; the findings are associational and hypothesis-generating.

## Introduction

Elite football performance depends on maintaining player availability, squad continuity, and physical readiness throughout increasingly congested competitive schedules. Injury burden represents a major disruptor of this performance ecosystem by reducing player availability and compromising collective training continuity, tactical preparation, and competitive performance outcomes (1, 2). Injury-related player unavailability necessitates modifications to training sessions, adjustments to match-day strategies, and changes in squad rotation, creating additional operational and economic challenges for professional clubs (3, 4). Accordingly, injuries should be viewed not merely as isolated medical events but as systemic disruptions that influence both sporting performance and long-term squad management (3). Against this background, understanding the epidemiology and burden of injuries in professional football has become essential for both performance optimization and evidence-based decision-making.

Professional football exposes players to a considerable risk of injury. Elite footballers sustain approximately 7–9 injuries per 1,000 h of exposure, with muscle and ligament injuries accounting for the greatest time-loss burden (5, 6). Beyond the direct loss of playing time, recurrent and severe injuries reduce training availability, disrupt periodization, and challenge squad management throughout the competitive season. Prospective football-specific surveillance provides complementary evidence on injury epidemiology: in a Colombian medical football tournament, lower-limb and muscle/tendon injuries were the most frequently reported categories, although injury estimates vary according to competition level, player population and surveillance method (7). Collectively, the cumulative injury burden compromises player availability and has important implications for team selection, squad rotation, and competitive performance across a season (8). In particular, recurrent muscle injuries are characterized by high recurrence rates and prolonged absences, whereas severe joint injuries often result in extended time away from training and competition, further reducing player availability (9–11). In this context, modifiable lower-extremity physical qualities are also relevant to player readiness and injury-prevention frameworks. In youth football players, an eight-week programme integrating balance and plyometric training improved static and dynamic balance, ankle mobility, lower-leg strength and jump performance (12). The trial did not assess injury incidence or player valuation, but it provides football-specific evidence that targeted physical preparation can improve capacities relevant to lower-extremity readiness. Although illness episodes generally cause shorter absences, they may affect multiple players simultaneously and temporarily disrupt squad availability during congested competition periods. Beyond their consequences for sporting performance, these disruptions also carry important economic implications by influencing how clubs assess a player’s current and future value.

Player market value represents one of the primary indicators of a footballer’s sporting and economic value, reflecting current performance, future potential, and career trajectory (13, 14). Accordingly, player valuation reflects a combination of age, on-field performance, contract characteristics, market conditions, and increasingly, factors affecting future availability such as injury history (14–16). Injury history is increasingly recognized as an important determinant of player valuation because it shapes expectations regarding future availability and performance capacity (16). Econometric evidence suggests that each additional day of injury-related absence is associated with approximately a 0.2% reduction in player valuation, while traumatic injuries have been linked to wage reductions of around 12% (15, 17). Despite these findings, most studies have considered injury as one of several explanatory variables within broader valuation models, whereas large-scale evidence on how specific injury characteristics relate to the pre- to post-injury change in market value remains scarce and methodologically heterogeneous (16, 18). Given that injuries generate substantial sporting and financial costs for professional clubs (19, 20), their direct association with player valuation remains poorly understood despite its clear practical importance.

Taken together, these gaps highlight the need for large-scale evidence on how injury characteristics relate to the change in a player’s market value after an injury, with explicit attention to the fact that the same player contributes several injury episodes and that valuation changes are dominated by age-related career dynamics. Therefore, this study aimed to quantify the association between injury duration and injury category and the pre- to post-injury change in market value in a large retrospective cohort of professional football players, adjusting for age, playing position, baseline value, season and league; to test whether these associations differ by age; and to compare injured players with matched injury-free players over the same period. By integrating sports medicine and football economics, the study provides association-level evidence that can inform hypotheses for injury management, player valuation and squad planning.

## Methods

### Study design

A retrospective cohort study of injury episodes was conducted to examine the association between injury characteristics and the subsequent change in market value among professional football players. The selection frame was the set of 2,624 players listed in the 2024 squads of the five major European top divisions (Premier League, LaLiga, Serie A, Bundesliga and Ligue 1); for each player the complete injury history and valuation history recorded on Transfermarkt were retrieved, so that injury episodes span July 2008 to August 2024 and occurred in the various leagues in which the players competed during their careers (only 170 episodes occurred in the Turkish top division). Because eligibility was defined by membership in a Big-Five league squad in 2024, the cohort represents retrospectively reconstructed injury histories of players who remained in or reached this elite selection frame by 2024, rather than a population-based cohort of all professional players active since 2008. The unit of analysis was the injury episode, and players could contribute several episodes. The study is reported in accordance with the STROBE guideline for cohort studies (21); the completed checklist is provided as Supplementary material.

### Data source

Player injury records and market-value histories were obtained from the publicly available Transfermarkt database (Transfermarkt GmbH, Hamburg, Germany), which provides dated valuation updates, injury diagnoses, injury start and end dates, and matches missed. The database was accessed in January 2025 using a custom Python-based web-scraping pipeline. An archived snapshot of the extracted data was used for all analyses reported here; a de-identified analysis dataset, the diagnosis dictionary, and the analysis code used to generate every table, figure, and numerical result are provided (see Data availability). Publicly available information was extracted exclusively for scientific research purposes, and only aggregated results are presented.

Transfermarkt records are not collected through a standardised prospective medical-surveillance system. Accordingly, the completeness and clinical specificity of recorded diagnoses and absence periods may vary according to player prominence, league, season, injury severity and the reporting practices of the underlying public sources. No clinical reference standard was available to quantify the sensitivity or specificity of these records.

### Data structure

The archived database contained 15,327 injury episodes recorded for the 2,624 squad players, of which 13,205 had both a valuation in effect before the injury and a valuation update after the injury (see Data extraction and processing). After removal of the 1st and 99th percentile tails of the outcome, the analytic dataset comprised 12,948 injury episodes from 2,061 players (median 5 episodes per player, maximum 15). Each observation combined the injury characteristics with the player’s age at injury, playing position, season, league at the time of the injury and the two market values.

### Participant selection

Players were eligible if they were listed in the 2024 squad of a club in one of the five major European top divisions. All injury episodes in their Transfermarkt injury history were eligible if a market value was in effect 120 days before the injury start and at least one valuation update was recorded after the 120 days following the injury end. Episodes without a post-injury valuation (n = 1,635), without a pre-injury valuation (n = 509) or without a recorded age (n = 282) were excluded, and 257 episodes with a percentage change below the 1st percentile (−69%) or above the 99th percentile (1,900%) were trimmed to limit the influence of extreme ratios arising primarily from very low pre-injury values. The full untrimmed distribution, a winsorised outcome, and the logarithm of the post-injury to pre-injury value ratio were examined in sensitivity analyses. Because inclusion required membership of a Big-Five league squad in 2024, the cohort is conditioned on players remaining in or reaching this elite selection frame. Players whose careers ended, declined substantially, or failed to reach this level following a severe injury are therefore under-represented. This selection mechanism may attenuate adverse injury–market-value associations and limits the generalisability of the findings to the broader population of professional football players (see Limitations).

### Data extraction and processing

For each injury episode, the diagnosis label, start and end dates, days lost and matches missed were retrieved together with the player’s dated valuation history. The pre-injury market value was defined as the valuation in effect 120 days before the injury start (i.e., the most recent valuation update on or before that date), and the post-injury market value as the first valuation update recorded after the 120-day period following the injury end. The 120-day thresholds were operational design choices intended to place both valuations outside the periods immediately surrounding injury onset and return from injury and to accommodate the irregular timing of Transfermarkt valuation updates. They should not be interpreted as clinically validated cut-offs. The study was not preregistered; however, these valuation rules were established during construction of the analysis dataset and were not selected by optimising the observed injury–valuation associations. The rules were verified against the complete valuation histories and reproduced the stored pre- and post-injury values for 100 and 99.3% of episodes, respectively. Age at injury was calculated as completed years between the date of birth and the injury start. Injury diagnoses were classified with an explicit diagnosis dictionary covering all 292 distinct Transfermarkt labels (Supplementary Table S1), which assigns each label to one of four main categories aligned with the consensus tissue-type classification for football injuries (22)—muscle/tendon, joint/ligament, illness and other—and to a sub-group; the 93 labels whose tissue could not be determined from the label alone are flagged in the dictionary. Data extraction, cleaning and coding were performed with a documented, fully scripted pipeline.

### Ethics statement

This study used publicly available data from the Transfermarkt database and involved no direct interaction with human participants or access to private health records. No additional personal data were collected or processed by the research team. Therefore, institutional ethics approval and informed consent were not required under applicable national regulations.

### Variables

#### Outcome

The primary outcome was the percentage change in market value, calculated as 100 × (post-injury value−pre-injury value)/pre-injury value, with the two values defined above. Positive values indicate an increase and negative values a decline. Percentage change was retained as the primary outcome because it is directly interpretable in football valuation terms and was used in the original analysis. However, percentage change can become highly skewed when the pre-injury value is low and is mathematically coupled to the pre-injury value through its denominator. To address these issues, the primary regression models adjusted for pre-injury market value, the 1st and 99th percentile tails of the percentage-change outcome were trimmed, and sensitivity analyses were performed using the untrimmed outcome, a winsorised outcome, and the logarithm of the post-injury to pre-injury value ratio. These approaches assess the robustness of the findings to extreme ratios and outcome scale, but they cannot completely eliminate regression to the mean or denominator-related coupling. Because the pre-injury valuation precedes the injury start by at least 120 days and the post-injury valuation follows the injury end by at least 120 days, the outcome captures the change between two scheduled Transfermarkt valuation updates that bracket the injury rather than an injury-specific reassessment (see Results, Valuation timing).

#### Exposures

The primary exposure variables were injury duration (days lost) and injury category. Injury duration was analysed as a continuous variable (per 30 days) and in four predefined groups (1–14, 15–30, 31–90, and >90 days; the 90th day belongs to the 31–90-day group). Injury category comprised muscle/tendon injuries, joint/ligament injuries, illness (including COVID-19) and other injuries, the last group containing contusions, fractures and bone stress injuries, spine and back conditions, head, face and neck injuries, lacerations, region-only or unknown diagnoses, unspecified surgery and non-clinical absences such as fitness, rest and quarantine (Supplementary Table S2). The “other” category was used as a residual category because several constituent diagnoses were too sparse for stable separate modelling and because some public-database labels did not identify a unique tissue diagnosis. It should therefore be interpreted as a heterogeneous data category rather than as a clinically homogeneous injury group. The complete composition of this category, including the number of episodes assigned to each sub-group, is provided in Supplementary Table S2.

#### Covariates

Prespecified covariates were age at injury (in years, centred at 25 years; four groups, <23, 23–27, 28–30 and >30 years, were used for descriptive analyses), playing position (13 Transfermarkt positions; reference centre-back), pre-injury market value (per EUR 1 million), season, and league group at the time of the injury (English, Spanish, Italian, German or French top division, other top division, second tier, cup, youth or regional competition, or unknown). Cumulative prior injury burden was not included as a covariate in the primary models. Player-clustered standard errors account for correlation among repeated episodes from the same player but do not control for confounding by previous injury history. The archived dataset did not contain consistently measured information on playing time, recent performance, contract duration, international status, club quality, transfer activity, or recent market-value trajectory for all episodes. These factors may influence both injury characteristics and subsequent market-value change and were therefore not included in the primary models. The adjusted estimates should consequently be interpreted as associations conditional on the available covariates rather than as injury-specific causal effects.

### Statistical analysis

All analyses were performed in Python 3.13 using pandas, NumPy, SciPy, statsmodels and Matplotlib (23–27); all tables, figures and numerical statements were generated from the analysis dataset by a single scripted pipeline. Because the outcome was heavy-tailed, continuous variables are summarised as median and interquartile range (IQR) together with mean and standard deviation (SD), and group means are accompanied by 95% confidence intervals from a player-cluster bootstrap (1,000 resamples of players) to respect the dependence between episodes of the same player.

Differences in market-value change across injury categories, duration groups and age groups were described with the Kruskal–Wallis test, reported with the effect size η^2^H = (H − k + 1)/(*n* − k), followed by all pairwise Dunn comparisons with Bonferroni correction within each family of six comparisons (28). Spearman correlations are reported with player-cluster bootstrap confidence intervals. Because these tests treat episodes as independent, they are presented as exploratory descriptions of the episode-level distribution, and they were repeated on the earliest episode of each player.

Primary inference relied on linear regression models with cluster-robust standard errors clustered by player (29), with t-based confidence intervals using the number of players minus one as degrees of freedom. Four models were fitted on the same complete-case sample: Model 1 (injury duration, injury category and age), Model 2 (Model 1 plus playing position), Model 3 (Model 1 plus pre-injury market value) and Model 4 (duration group, injury category, age, playing position, pre-injury value, season and league group). The three models of the original submission were also re-estimated with conventional and player-clustered standard errors (Supplementary Table S3). Model 5 added age × injury category and age × duration group interaction terms to Model 4; each interaction family was tested with a joint Wald F test of all its coefficients, with Holm adjustment across the two families, and adjusted category and duration differences were computed at selected ages by standardisation over the observed covariate distribution. A linear mixed model with a random intercept for player (restricted maximum likelihood) was fitted with the Model 4 covariates to quantify the intraclass correlation of episodes within players.

Sensitivity analyses repeated Model 4 using only the earliest recorded injury episode from each player, on the untrimmed sample and with a winsorised outcome, with the logarithm of the value ratio as outcome, with the original valuation-record age variable, with the original keyword-based injury classification, with one episode retained per unique pre/post valuation pair to assess the influence of overlapping injury episodes assigned to the same valuation interval, and with a start-anchored ±120-day valuation specification that did not allow the measurement interval to increase with injury duration. Additional analyses modelled age as groups or as a B-spline and excluded non-clinical and unknown diagnoses (Supplementary Table S9). Restriction to the earliest recorded episode reduced the influence of repeated documented injury episodes within the archived dataset, although it did not provide a direct adjustment for cumulative prior injury burden or injuries that may have occurred before the observable record. The timing of the valuations relative to the injury was described by the number of days between the injury start and the pre-injury valuation and between the injury end and the post-injury valuation.

Finally, an exploratory matched-control comparison assessed differences in log-value change between injured players and injury-free player-periods over the same calendar dates. For each injury episode, a control player was sought from the same 2024 squad league and position group, aged within 1 year of the injured player at injury start, with no recorded injury episode between 120 days before injury start and 120 days after injury end, and with a pre-injury market value within 0.20 log units of that of the injured player (nearest-neighbour 1:1 matching with replacement). The control’s pre- and post-index values were selected using the same valuation rules and calendar dates as for the corresponding injured episode. Consequently, each injured player and matched control was evaluated over the same calendar interval. This design was intended to account for age-related, market-wide and calendar-period changes occurring over the longer observation intervals associated with prolonged injuries, but it did not control for player-specific changes in performance, contract status, transfer activity or other unmeasured time-varying characteristics. Controls could be reused across matched episodes. In addition, control eligibility was assessed only through 120 days after the index injury end and did not require controls to remain free of a recorded injury until the actual post-index valuation date. The estimand was the injured-minus-control difference in log(post-injury value/pre-injury value), summarised as a mean with player-clustered confidence intervals overall and by injury category, duration group and age group. Balance on the measured matching variables was assessed using standardised mean differences for age, baseline value and valuation timing. The difference in value trend during the 360 days before the pre-index valuation was examined as a limited pre-trend diagnostic, and the matching rule was relaxed in three sensitivity specifications. Because a single pre-trend diagnostic cannot establish parallel trends or balance in unmeasured characteristics, this analysis was interpreted as an exploratory matched-control comparison rather than a causal difference-in-differences design. Statistical significance was set at *p* < 0.05 (two-sided).

## Results

### Sample characteristics

The analytic dataset comprised 12,948 injury episodes from 2,061 players (median 5 episodes per player, maximum 15). The mean age at injury was 24.4 years (SD 3.8). The median injury duration was 16 days (IQR 8 to 35; mean 31.5, SD 44.6), indicating a right-skewed distribution driven by a smaller proportion of prolonged episodes. The median market-value change was 0.0% (IQR − 25.0 to 66.7), whereas the mean was 64.7% (SD 200.9); 41.3% of episodes showed a decrease, 10.1% no change and 48.6% an increase. Table 1 summarises the outcome by injury category, duration group and age group.

**Table 1:** Market-value change (%) by (A) injury category, (B) injury-duration group and (C) age group.

(A) Injury category							
Injury category	Episodes	Players	Mean (95% CI)	SD	Median (IQR)	Decrease/no change/increase (%)	Duration, median (mean) days
Muscle/tendon	4,698	1,463	55.0 (48.7 to 61.5)	189.7	0.0 (−25.0 to 55.6)	44/10/46	19 (29.5)
Joint/ligament	2,594	1,295	69.0 (60.8 to 76.9)	200.8	6.7 (−23.1 to 66.7)	40/10/50	29 (54.0)
Illness	1,732	1,023	74.1 (63.2 to 85.1)	224.2	3.9 (−22.2 to 69.2)	40/10/50	8 (11.8)
Other	3,924	1,515	69.4 (61.6 to 77.0)	202.9	0.0 (−22.2 to 75.0)	40/10/50	14 (27.8)
Total	12,948	2,061	64.7 (60.0 to 69.6)	200.9	0.0 (−25.0 to 66.7)	41/10/49	16 (31.5)

(B) Injury-duration group							
Duration group (days)	Episodes	Players	Mean (95% CI)	SD	Median (IQR)	Decrease/no change/increase (%)	Duration, median (mean) days
1–14	5,795	1,629	67.9 (61.4 to 74.6)	205.0	6.7 (−20.0 to 66.7)	39/10/51	8 (7.8)
15–30	3,387	1,417	61.7 (54.7 to 69.3)	191.7	0.0 (−25.0 to 63.6)	42/10/48	20 (21.0)
31–90	2,832	1,402	59.7 (52.7 to 67.0)	186.6	0.0 (−25.0 to 62.5)	43/11/47	46 (50.6)
>90	934	690	71.7 (55.8 to 90.1)	245.3	0.0 (−33.3 to 60.0)	47/11/42	134 (159.3)
Total	12,948	2,061	64.7 (60.0 to 69.6)	200.9	0.0 (−25.0 to 66.7)	41/10/49	16 (31.5)

(C) Age group							
Age group (years)	Episodes	Players	Mean (95% CI)	SD	Median (IQR)	Decrease/no change/increase (%)	Duration, median (mean) days
<23	4,407	1,421	156.2 (146.0 to 166.0)	291.8	50.0 (0.0 to 177.8)	21/9/70	18 (35.7)
23–27	5,835	1,389	33.6 (29.3 to 37.9)	118.2	0.0 (−22.2 to 50.0)	42/10/47	15 (30.4)
28–30	1,839	559	−12.4 (−15.5 to −9.0)	48.4	−22.2 (−40.0 to 0.0)	69/11/20	15 (27.8)
>30	855	231	−27.0 (−30.1 to −23.8)	32.3	−33.3 (−50.0 to −14.3)	82/10/8	15 (25.7)
Total	12,948	2,061	64.7 (60.0 to 69.6)	200.9	0.0 (−25.0 to 66.7)	41/10/49	16 (31.5)

Episodes, injury episodes; Players, distinct players contributing to the group (a player can appear in several groups, so player counts do not sum to 2,061). Mean 95% CI from a player-cluster bootstrap (1,000 resamples). Market-value change, 100 × (post-injury value−pre-injury value)/pre-injury value, where the pre-injury value is the valuation in effect 120 days before the injury start and the post-injury value is the first valuation update after 120 days following the injury end. Age, completed years at injury start.

### Injury category and market value change

Market-value change differed across injury categories (*H*(3) = 34.35, *n* = 12,948, *p* < 0.001, η^2^H = 0.0024), but the effect size was negligible. The median change was 0.0% for muscle/tendon injuries, 6.7% for joint/ligament injuries, 3.9% for illness and 0.0% for the ‘other’ category (Table 1A; Figure 1). In Dunn pairwise comparisons only the contrasts involving muscle/tendon injuries were significant (Supplementary Table S5), and the category difference was not present when only the earliest episode of each player was analysed (H = 2.72, *p* = 0.437). The composition of the ‘other’ category is given in Supplementary Table S2 and Figure S1. This residual category comprised 3,924 episodes (30.3% of the analytic sample) and included clinically heterogeneous diagnoses and non-clinical absences; it was therefore not interpreted as representing a common injury mechanism.

**Figure 1:**
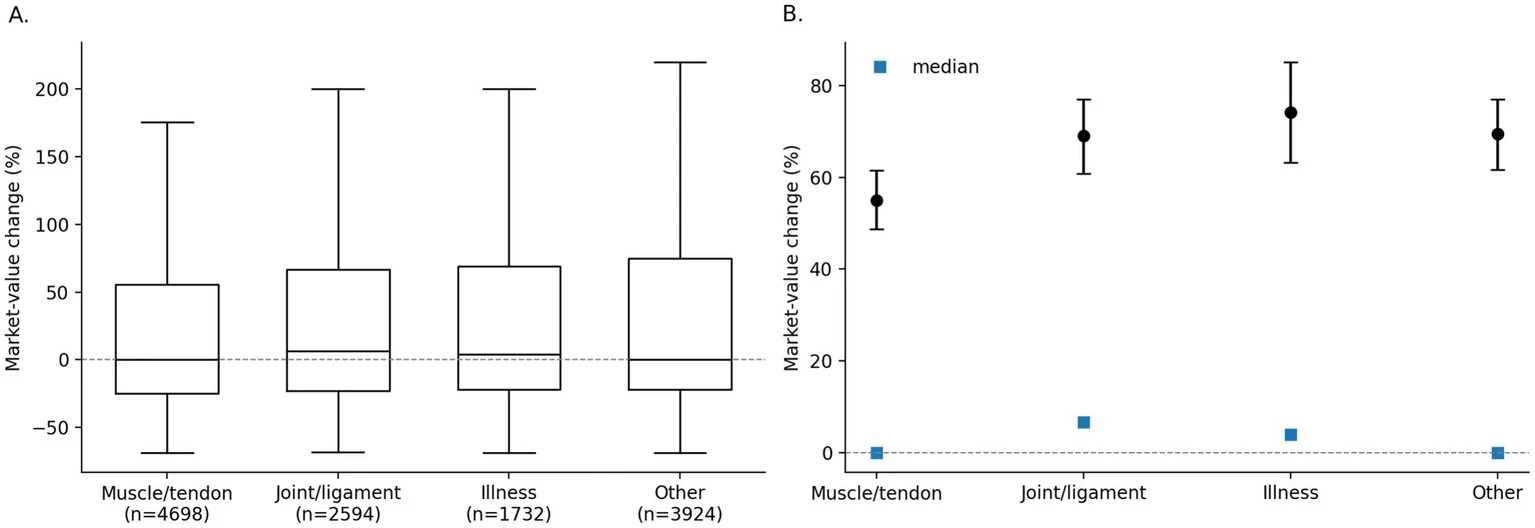
Market-value change (%) by injury category. **(A)** Median and interquartile range (outliers not drawn); **(B)** mean with 95% player-cluster bootstrap confidence interval (circles) and median (squares). n, injury episodes.

### Injury duration strata and market value change

Market-value change differed across injury-duration groups (*H*(3) = 36.01, n = 12,948, *p* < 0.001, η^2^*H* = 0.0025), again with a negligible effect size. Mean changes were 67.9, 61.7, 59.7, and 71.7% for episodes of 1–14, 15–30, 31–90, and >90 days, but the medians were 6.7, 0.0, 0.0 and 0.0% (Table 1B; Figure 2), so that the ordering of the means reflects extreme values rather than the typical episode. In Dunn comparisons, episodes of 1–14 days differed from every longer group and 15–30 days differed from >90 days, whereas 15–30 vs. 31–90 and 31–90 vs. > 90 did not (Supplementary Table S6). The duration difference persisted in the earliest-episode sample (*p* = 0.003).

**Figure 2:**
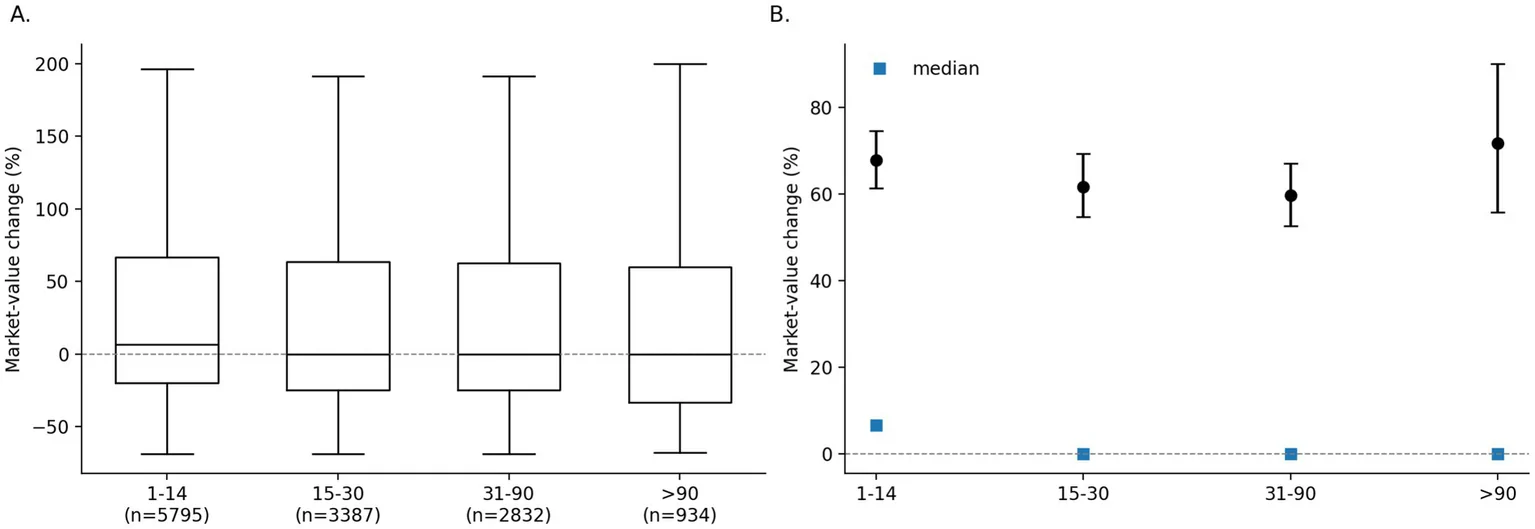
Market-value change (%) by injury-duration group. **(A)** Median and interquartile range (outliers not drawn); **(B)** mean with 95% player-cluster bootstrap confidence interval (circles) and median (squares). n, injury episodes.

### Age group differences

Age was strongly associated with market-value change (*H*(3) = 2873.07, *n* = 12,936, *p* < 0.001, η^2^*H* = 0.222). The median change was 50.0% for players younger than 23 years, 0.0% for 23–27 years, −22.2% for 28–30 years and −33.3% for players older than 30 years (Table 1C; Figure 3), and all six pairwise comparisons were significant (Supplementary Table S7).

**Figure 3:**
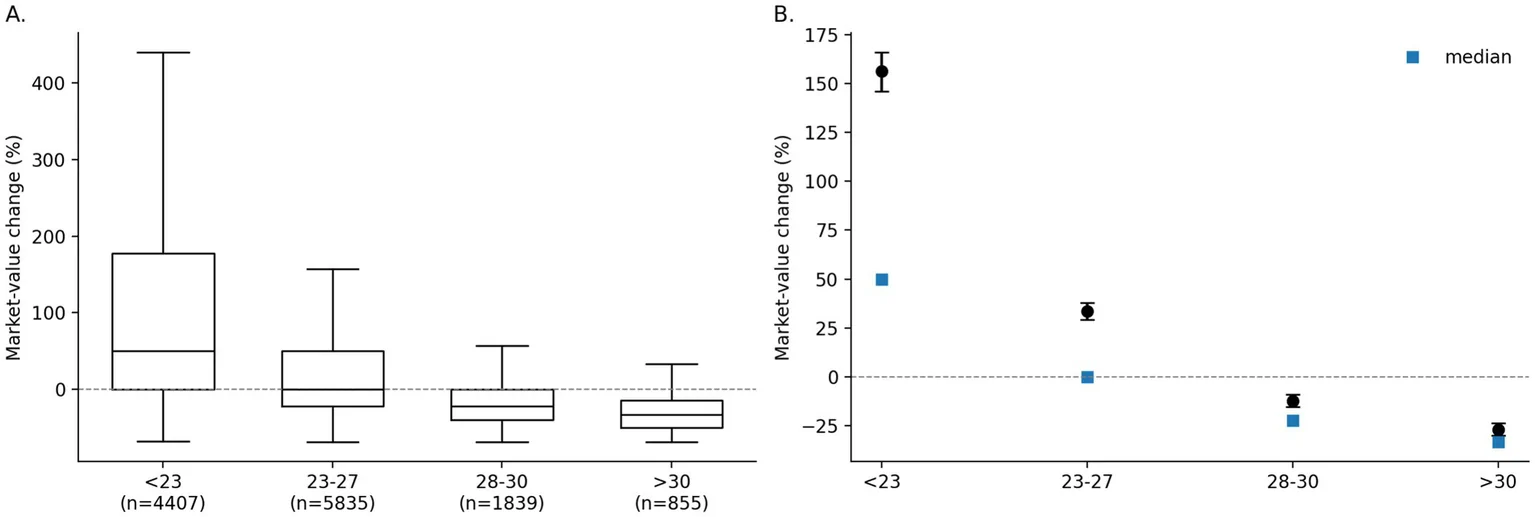
Market-value change (%) by age group (completed years at injury start). **(A)** Median and interquartile range (outliers not drawn); **(B)** mean with 95% player-cluster bootstrap confidence interval (circles) and median (squares). n, injury episodes.

### Correlation structure among key variables

Injury duration showed a weak negative correlation with market-value change (Spearman *ρ* = −0.051; player-cluster bootstrap 95% CI −0.071 to −0.031). Age (*ρ* = −0.489) and pre-injury market value (*ρ* = −0.365) showed the strongest bivariate correlations among the numeric variables examined (Figure 4).

**Figure 4:**
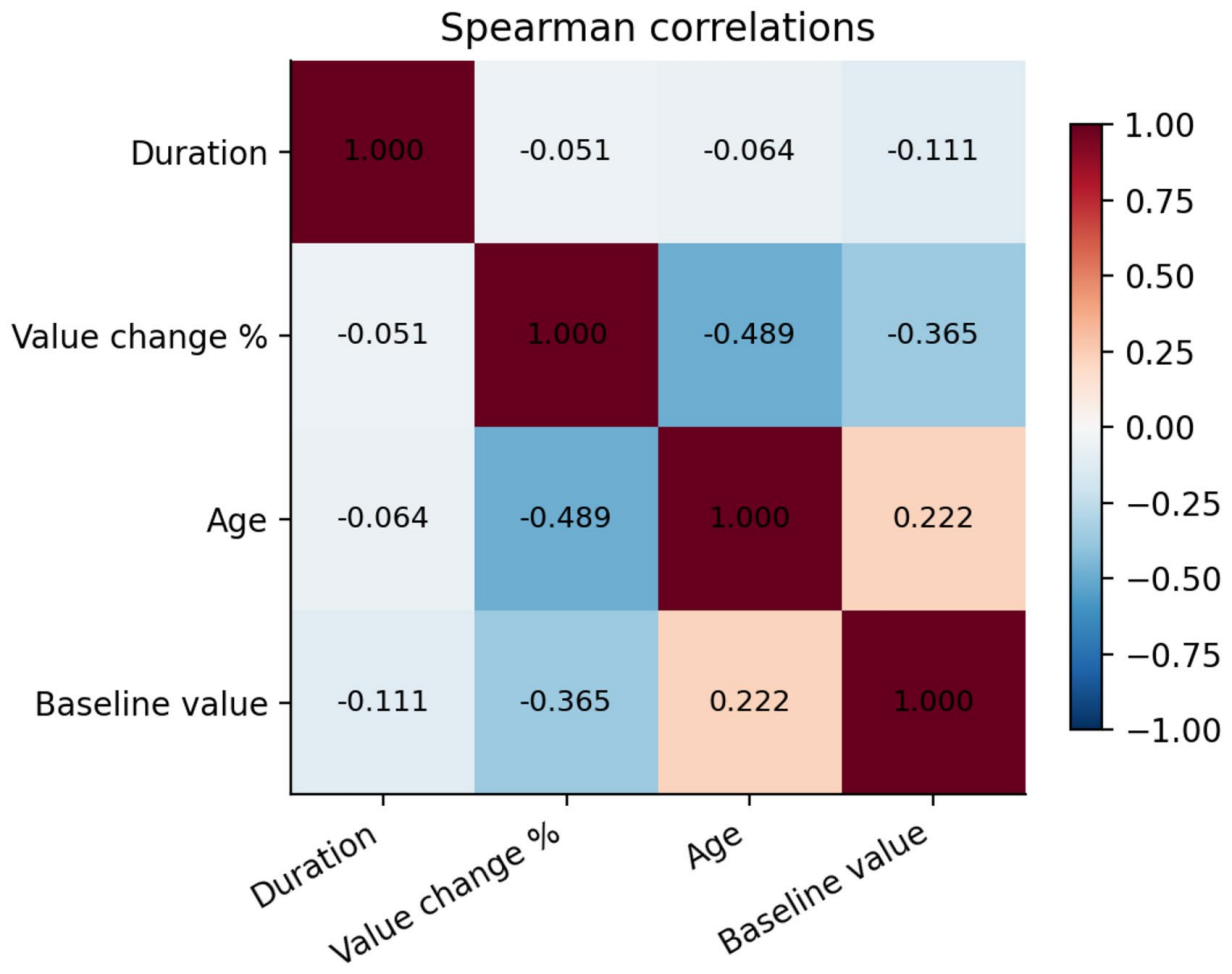
Spearman correlation matrix of injury duration, market-value change, age at injury and pre-injury market value.

### Regression models

Table 2 shows the coefficients of Models 1–4 with player-clustered 95% confidence intervals. Re-estimating the originally submitted models with player-clustered standard errors left the coefficients unchanged and increased the standard errors by a factor of 1.07–1.09 for the injury-category terms, 1.48–1.54 for age and 1.98 for pre-injury value (Supplementary Table S3). In the fully adjusted Model 4 (n = 12,936 episodes from 2,059 players; *R*^2^ = 0.177, adjusted *R*^2^ = 0.174, leaving 82.3% of the variance unexplained), substantial variation in market-value change remained unexplained by the available injury, demographic and competition-level variables. Each additional year of age was associated with −18.2 (95% CI -19.7 to −16.8; *p* < 0.001) percentage points and each additional EUR 1 million of pre-injury value with −1.9 (95% CI -2.3 to −1.6; *p* < 0.001) percentage points. Compared with episodes of 1–14 days, episodes of 15–30, 31–90 and >90 days were associated with −11.4 (95% CI -19.6 to −3.2; *p* = 0.006), −23.6 (95% CI -32.4 to −14.8; *p* < 0.001) and −26.4 (95% CI -42.6 to −10.1; *p* = 0.002) percentage points, respectively. Compared with muscle/tendon injuries, joint/ligament injuries (−5.4 (95% CI -14.4 to 3.5; *p* = 0.231)), illness (1.0 (95% CI -10.4 to 12.3; *p* = 0.865)) and the ‘other’ category (−8.9 (95% CI -17.1 to −0.7; *p* = 0.034)) did not differ materially, and the small ‘other’ difference disappeared when non-clinical and unknown diagnoses were excluded (−1.4 (95% CI -11.0 to 8.3; *p* = 0.779); Supplementary Table S9). A random-intercept model gave an intraclass correlation of 0.28 for episodes within players, with fixed effects concordant with Model 4 (Supplementary Table S8).

**Table 2:** Linear regression of market-value change (percentage points) on injury characteristics: coefficients with player-clustered 95% confidence intervals (Models 1–4).

Term	Model 1	Model 2	Model 3	Model 4
Intercept	57.0 (50.6 to 63.4); <0.001	65.1 (54.0 to 76.1); <0.001	91.4 (83.0 to 99.7); <0.001	114.4 (95.8 to 133.0); <0.001
Injury category: Illness	6.4 (−4.7 to 17.4); 0.259	4.1 (−7.0 to 15.2); 0.468	1.3 (−9.5 to 12.1); 0.816	1.0 (−10.4 to 12.3); 0.865
Injury category: Joint/ligament	1.2 (−8.0 to 10.4); 0.793	0.3 (−8.9 to 9.5); 0.957	−2.3 (−11.3 to 6.8); 0.622	−5.4 (−14.4 to 3.5); 0.231
Injury category: Other	2.4 (−5.7 to 10.5); 0.564	0.3 (−7.8 to 8.5); 0.942	−5.1 (−13.1 to 3.0); 0.216	−8.9 (−17.1 to −0.7); 0.034
Injury duration (per 30 days)	−4.5 (−6.9 to −2.0); <0.001	−4.8 (−7.3 to −2.4); <0.001	−6.1 (−8.5 to −3.8); <0.001	—
Age (per year, centred at 25)	−18.8 (−20.2 to −17.5); <0.001	−19.4 (−20.7 to −18.0); <0.001	−18.3 (−19.6 to −17.0); <0.001	−18.2 (−19.7 to −16.8); <0.001
Pre-injury market value (per EUR 1 million)	—	—	−2.0 (−2.3 to −1.7); <0.001	−1.9 (−2.3 to −1.6); <0.001
Duration group: 15–30	—	—	—	−11.4 (−19.6 to −3.2); 0.006
Duration group: 31–90	—	—	—	−23.6 (−32.4 to −14.8); <0.001
Duration group: >90	—	—	—	−26.4 (−42.6 to −10.1); 0.002
Episodes/players	12,936/2,059	12,936/2,059	12,936/2,059	12,936/2,059
*R*^2^/adjusted *R*^2^	0.1241/0.1238	0.1271/0.1260	0.1619/0.1615	0.1772/0.1744

All models were fitted on the same 12,936 episodes from 2,059 players; standard errors are clustered by player (CR1) with t-based confidence intervals. Reference categories: muscle/tendon injury; 1–14 days; centre-back; season 2023/24; English top division. Age is centred at 25 years; injury duration is per 30 days in Models 1–3 and in groups in Model 4; pre-injury value is per EUR 1 million. Coefficients for playing position, season and league group are given in Supplementary Table S3b.

### Age interactions

Model 5 provided no evidence that the associations of injury category or injury duration with market-value change differed by age: age × injury category *F*(3, 2058) = 0.13, *p* = 0.940 (Holm-adjusted *p* = 0.940), age × duration group *F*(3, 2058) = 1.92, *p* = 0.125 (Holm-adjusted *p* = 0.249), and all six interaction terms jointly *F*(6, 2058) = 1.19, *p* = 0.310; *R*^2^ changed from 0.1772 in Model 4 to 0.1779 in Model 5 (Table 3). Figure 5 shows the adjusted category and duration differences across ages.

**Table 3:** Age interactions (Model 5): joint Wald tests and interaction coefficients.

Term	Coefficient/statistic	95% CI	*p*	Holm *p*
Joint test: age × injury_category	*F*(3, 2058) = 0.13	—	0.940	0.940
Joint test: age × duration_group	F(3, 2058) = 1.92	—	0.125	0.249
Joint test: all six interaction terms	*F*(6, 2058) = 1.19	—	0.310	—
Age × Illness	−0.56	−3.94 to 2.82	0.746	—
Age × Joint/ligament	−0.43	−3.25 to 2.38	0.762	—
Age × Other	−0.83	−3.42 to 1.75	0.527	—
Age × 15–30	0.44	−2.14 to 3.02	0.740	—
Age × 31–90	2.03	−0.73 to 4.80	0.149	—
Age × > 90	−4.00	−9.12 to 1.12	0.126	—

Model 5, Model 4 plus age × injury category and age × duration group (same 12,936 episodes, 2,059 players; player-clustered covariance). Joint tests evaluate all coefficients of an interaction family; Holm adjustment across the two families. Interaction coefficients are the change in the category or duration difference (percentage points) per additional year of age.

**Figure 5:**
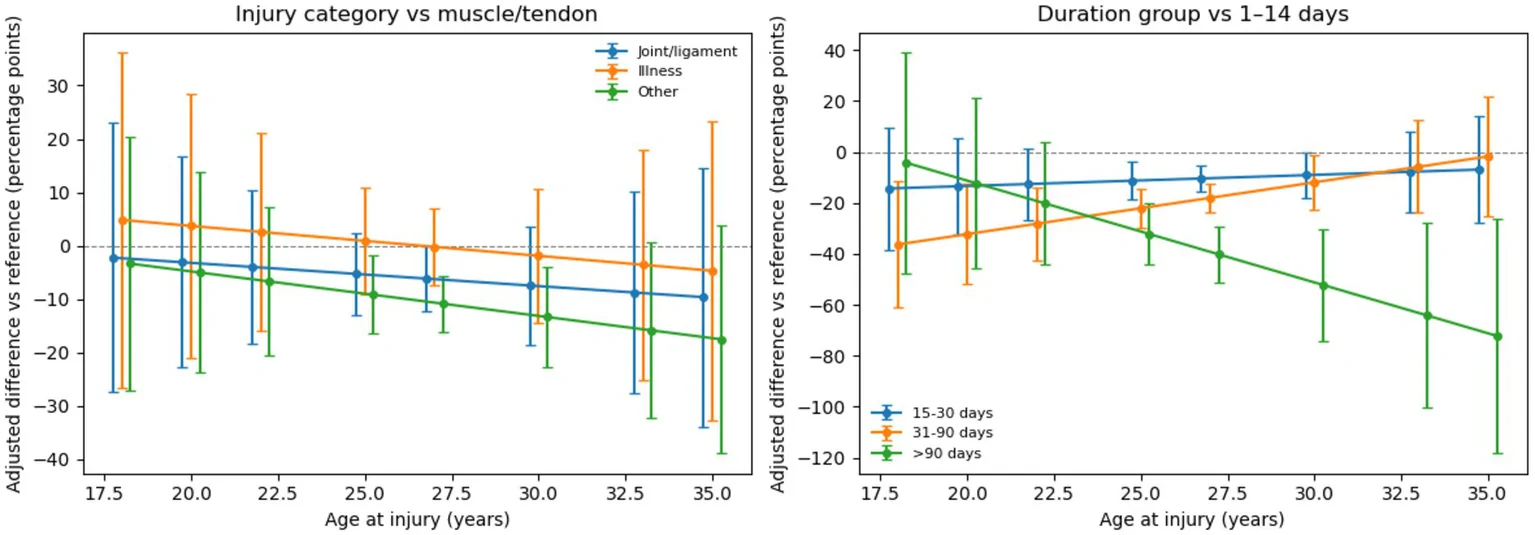
Adjusted differences in market-value change (percentage points) by age from Model 5: injury categories versus muscle/tendon injuries (left) and duration groups versus 1–14 days (right), with player-clustered 95% confidence intervals. Estimates at ages with fewer than 30 supporting episodes are extrapolations.

### Valuation timing

The pre-injury valuation was issued a median of 191 days before the injury start (IQR 152 to 240) and the post-injury valuation a median of 195 days after the injury end (IQR 157 to 242); the interval between the two valuations was a median of 416 days overall, 383 days for episodes of 1–14 days and 546 days for episodes longer than 90 days (Supplementary Table S13). Thus, the primary valuation interval increased mechanically with injury duration, confirming that the unadjusted and primary episode-level duration associations may partly reflect changes occurring over a longer observation period. In total, 3,202 episodes shared their valuation pair with another episode of the same player. Restricting the analysis to one episode per unique valuation pair left 11,223 episodes. The alternative start-anchored ±120-day valuation specification included 7,389 episodes and produced duration coefficients in the same direction as the primary analysis (Supplementary Table S9). Thus, the direction of the duration associations was not dependent on the primary injury-end-anchored post-injury valuation rule, although the irregular timing of Transfermarkt valuation updates precluded the use of exactly equidistant observations for every episode.

### Matched-control analysis

A total of 6,689 of 12,948 episodes (51.7%) were matched to an injury-free player-period; the remaining episodes had no control within the value caliper (n = 6,133) or did not meet the valuation-timing criteria. Matched pairs were well balanced on the measured matching variables of age, pre-injury value and valuation timing, with standardised mean differences below 0.01 (Supplementary Table S11). This balance does not demonstrate comparability in unmeasured performance, contract, transfer or injury-history characteristics. The injured-minus-control difference in log(post/pre) was −0.045 (95% CI − 0.070 to −0.020; *p* < 0.001), equivalent to 4.4% (95% CI − 6.7 to −2.0%) lower value growth for injured players. The pre-index trend contrast over the preceding 360 days was −0.018 (95% CI − 0.051 to 0.015). Although this estimate did not indicate a clear difference, its confidence interval remained compatible with modest pre-existing differences and a single diagnostic cannot establish the parallel-trends assumption. The difference increased with injury duration (1–14 days: 2.1% (95% CI -1.2 to 5.6); 15–30 days: −5.7% (95% CI -9.7 to −1.4); 31–90 days: −7.5% (95% CI -11.3 to −3.5); >90 days: −26.9% (95% CI -32.9 to −20.5); heterogeneity *p* < 0.001) but did not differ between injury categories (*p* = 0.215) (Figure 6). Relaxing the matching rule increased the matched share to 76–97% with overall differences between −5.9% and −6.5% (Supplementary Table S10).

**Figure 6:**
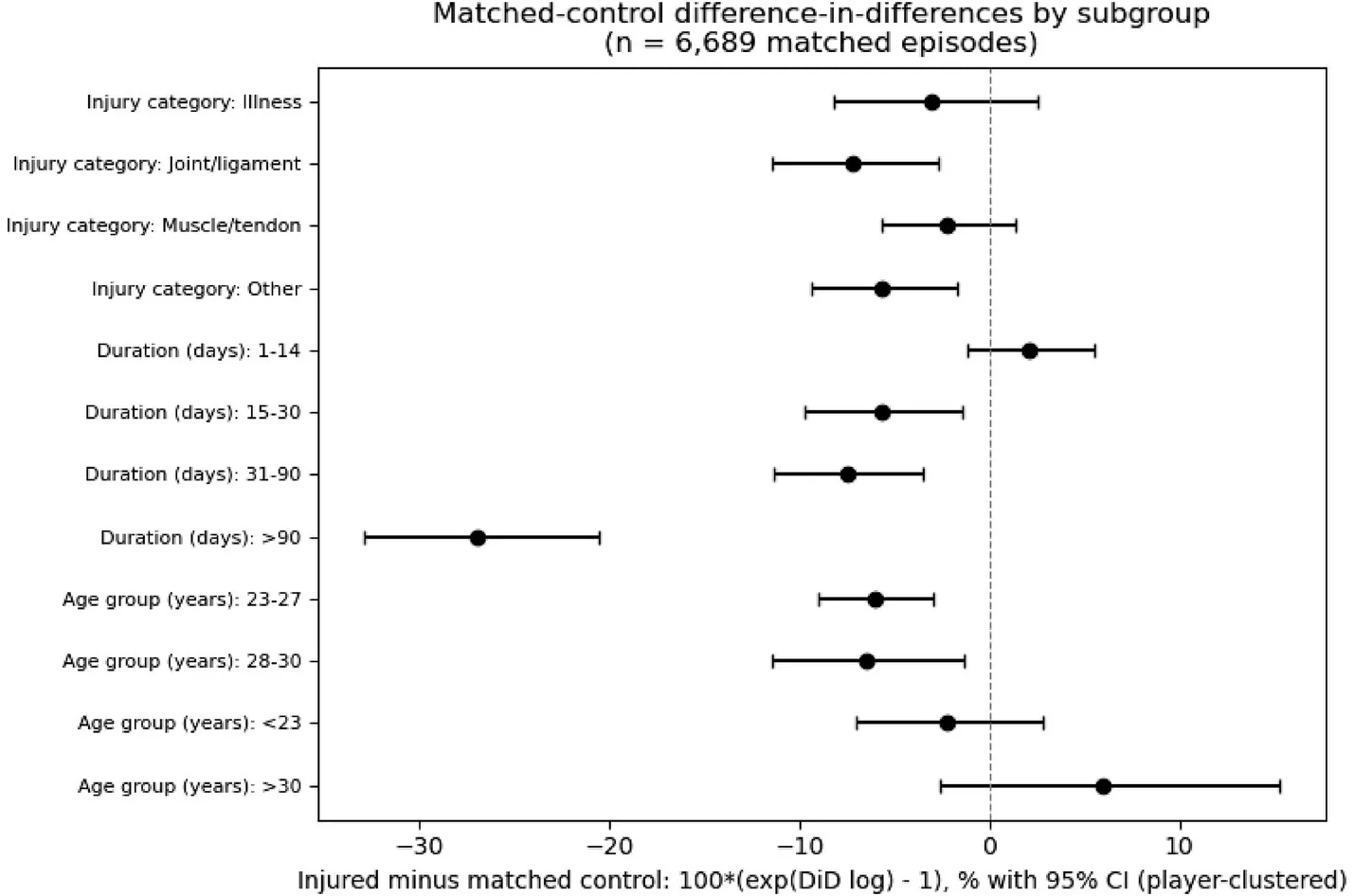
Exploratory matched-control comparison of log-value change: injured minus matched control difference in log(post-injury value/pre-injury value), expressed as a percentage, by injury category, duration group and age group, with player-clustered 95% confidence intervals (n = 6,689 matched episodes).

### Sensitivity analyses

The direction and approximate magnitude of the duration associations were maintained across all sensitivity analyses (Supplementary Table S9); confidence intervals were widest in the untrimmed sample and in the earliest-episode sample. The duration-group difference also remained evident when only the earliest recorded episode from each player was analysed. This restriction reduced the influence of repeated documented injuries within players but did not directly adjust for cumulative prior injury burden. Using the original age variable or the original keyword-based injury classification did not change the conclusions.

## Discussion

This retrospective cohort study of 12,948 injury episodes from 2,061 professional football players provides large-scale evidence on the association between injury characteristics and the change in market value between the valuations that bracket an injury. Four findings stand out. First, differences in market-value change between injury categories and between duration groups were statistically significant but negligible in size, and the category difference did not survive restriction to one episode per player, whereas age accounted for a large share of the variation. Second, after adjustment for age, position, pre-injury value, season and league, and with standard errors that account for repeated episodes of the same player, injuries lasting more than 14 days were associated with a lower percentage change than shorter injuries, with the largest differences for injuries of 31–90 days and of more than 90 days; injury category was not associated with the change. Third, there was no evidence that these associations differed by age. Fourth, in the matched-control analysis injured players showed a modest injury-related shortfall in value growth relative to injury-free players of the same age, position, league and baseline value, which increased monotonically with injury duration. These findings address a gap in the sports medicine and football economics literature by quantifying injury–valuation associations that had been proposed but inadequately measured (17, 30, 31), while showing that most of the variation in valuation change is unrelated to injury characteristics.

Our finding that longer injuries are associated with a lower subsequent change in market value is consistent with econometric evidence that each day of medical absence is associated with an approximately 0.2% reduction in player value (17) and with evidence that injury risk is priced into valuations (16). Two features of the present data qualify the interpretation. First, the pre-injury and post-injury valuations are scheduled Transfermarkt updates that precede and follow the injury by several months. The median interval between the two valuations increased from 383 days for injuries lasting 1–14 days to 546 days for injuries lasting more than 90 days. Consequently, part of the duration association in the primary episode-level models may reflect ageing, performance development, contract progression, transfer activity or broader market changes occurring over a longer observation period rather than injury duration itself. The direction of the duration associations was nevertheless maintained when a start-anchored ±120-day valuation specification was used. Second, the matched-control comparison applied the same calendar index dates and valuation rules to an injury-free player-period matched on league, position group, age and baseline value. This comparison therefore accounts for changes shared by similarly valued players over the same calendar interval and continued to show a duration-dependent shortfall. This comparison cannot establish that the observed difference was caused by injury because controls could be reused, their injury-free status was not required to extend to the actual post-index valuation date, and matching did not balance unmeasured performance, contract or transfer-related characteristics. The findings should therefore be interpreted as duration-related associations rather than as isolated causal effects of injury duration. In contrast, the pattern previously described as an intermediate-duration ‘value-retention risk window’ was not supported by the regenerated data: the medians of all groups lasting more than 14 days were zero, and the ordering of the group means was driven by extreme values.

Market-value change differed only marginally between injury categories. Muscle/tendon injuries had the lowest median change, and Dunn comparisons identified differences only between muscle/tendon injuries and the other three categories; however, the difference disappeared when only the earliest episode of each player was analysed and in the adjusted models, and the matched-control differences did not differ between categories. Pulici et al. (6) reported that muscle and ligament injuries account for the greatest exposure-adjusted time-loss burden in professional football; our results suggest that, once duration, age and baseline value are taken into account, the type of injury adds little information about the subsequent valuation change. The ‘other’ category comprised 3,924 episodes (30.3%) and intentionally combined sparse or incompletely specified labels, including contusions, fractures and bone stress injuries, spine and back conditions, head, face and neck injuries, region-only or unknown diagnoses, unspecified surgery, and non-clinical absences. It should therefore not be interpreted as a single injury mechanism. Reassuringly, the small adjusted difference observed for this category disappeared after non-clinical and unknown diagnoses were excluded, although the residual diagnostic heterogeneity remains a limitation.

Age showed a clear association with market-value change across the descriptive and adjusted analyses: players younger than 23 years showed a median increase of 50.0%, whereas players older than 30 years showed a median decline of 33.3%. Accordingly, a positive pre- to post-injury change should not be interpreted as an increase caused by the injury; rather, it may reflect the underlying appreciation of a player’s market value over the valuation interval, particularly among younger players. This pattern is consistent with human capital and life-cycle models in which younger players are appreciating assets with unrealised developmental potential and older players are subject to progressive depreciation (14, 15, 17). This pattern is consistent with human capital and life-cycle models in which younger players are appreciating assets with unrealised developmental potential and older players are subject to progressive depreciation (14, 15, 17). Although Carrieri et al. (15) reported that traumatic injuries reduced players’ wages, their analyses did not account for age-specific effects. Importantly, formal tests of age × injury category and age × duration interactions did not support the hypothesis that injury-related differences vary with age, and the adjusted duration differences were similar across the ages with adequate data support. The age association therefore describes the career-stage trajectory on which injuries are superimposed rather than an age-specific injury effect, and it underlines the need to adjust for age in any analysis of injury-related valuation change.

Although the present study examined valuation dynamics at the individual player level, its findings also provide insights into the broader sporting and economic consequences of injuries for professional clubs. Recent economic analyses have demonstrated the substantial financial burden of injuries, with English Premier League clubs losing an estimated £45 million per season because of injury-related performance decrements (19), while muscular injuries alone have been estimated to cost €365,811 per month in LaLiga (20). Our findings suggest that these financial consequences are unlikely to be uniformly distributed across injuries: the matched-control analysis indicates that the injury-related shortfall in value growth is concentrated in long-duration injuries, whereas the valuation change after short injuries was indistinguishable from that of injury-free players.

## Limitations

Several limitations should be acknowledged. First, Transfermarkt valuations are crowd-sourced estimates rather than realised transfer fees, although they correlate strongly with actual fees (17, 18). Second, the outcome spans two scheduled valuation updates, and the interval increased mechanically with injury duration. Longer intervals allow market value to change because of ageing, performance, contract progression, transfers and broader market conditions. The alternative valuation-window and matched-control analyses were directionally consistent, but neither can fully separate injury duration from unmeasured player-specific changes. Moreover, 24.7% of episodes shared an exact pre/post valuation pair with another injury episode from the same player. Although the one-episode-per-pair analysis was directionally consistent, market-value change cannot be uniquely attributed to a single injury episode. Third, the cohort was restricted to players who remained in or reached a Big-Five league squad in 2024. Players whose careers ended, declined substantially, or failed to reach this level after severe injury are under-represented; this selection mechanism may attenuate adverse injury–market-value associations and limits generalisability. Fourth, the matched-control analysis was exploratory: only about half of episodes were matched under the strict rule, controls could be reused, and the absence of a Transfermarkt record does not establish absence of injury. Control injury-free status was verified only through 120 days after the index injury end, not necessarily until the actual post-index valuation date, and unmeasured differences between groups may remain. Fifth, the primary models did not include cumulative prior injury burden. The public database also lacked standardised clinical information on recurrence and rehabilitation, as well as consistent data on playing time, performance, contract duration, international status, club quality, transfer activity and valuation trajectory. Unlike prospective surveillance systems using structured injury definitions and follow-up (7), Transfermarkt diagnoses are free-text public records (32); 93 of 292 labels could not be assigned a tissue type unambiguously. The heterogeneous ‘other’ category comprised 30.3% of episodes and included uncertain and non-clinical labels. Although clustered standard errors and earliest-episode analyses reduced the influence of repeated observations, residual confounding by prior injury history and diagnostic misclassification remain possible. Sixth, percentage change was heavy-tailed and mathematically coupled to pre-injury value. Trimming removed 257 extreme observations, and the untrimmed, winsorised and log-value-ratio analyses were directionally consistent; however, these procedures cannot eliminate regression-to-the-mean or denominator-related effects. Finally, the fully adjusted model explained 17.7% of the variance, and the intraclass correlation of 0.28 indicates substantial stable player-level variation. The findings therefore describe population-level associations and cannot support causal claims or individual prediction.

## Practical implications

The findings are hypothesis-generating rather than a basis for operational rules. They suggest that the duration of an injury, not its category, is the characteristic most consistently associated with a subsequent shortfall in value growth, and that this shortfall becomes economically meaningful only for long absences. Injury-prevention and return-to-play programmes that shorten absences may therefore have economic as well as sporting benefits, a hypothesis that requires controlled designs with performance and contract data. Because age dominates the valuation change and no age-specific injury effect was found, injury-related valuation analyses should adjust for age, but the present data do not support age-specific injury-management strategies.

## Conclusion

In this large retrospective cohort, the change in market value after an injury was dominated by the player’s age and pre-injury value. After adjustment, and with inference that accounts for repeated episodes within players, longer injuries were associated with a lower pre- to post-injury change and with a modest, duration-dependent shortfall in value growth relative to matched injury-free players, whereas injury category was not associated with the change and no age-by-injury interaction was found. These association-level findings, obtained with a fully reproducible pipeline, extend the understanding of the link between injury burden and player valuation and identify injury duration as the characteristic that deserves attention in future controlled studies integrating sports medicine and football economics.

## Data Availability

The datasets presented in this study can be found in online repositories. The names of the repository/repositories and accession number(s) can be found at: Kaggle–Football Player Injuries & Market Value Dataset: https://www.kaggle.com/datasets/gumush/football-player-injuries-market-value.
