# Supplementary material for: Injury characteristics and pre- to post-injury market-value change in professional football: a retrospective cohort study of 12,948 injury episodes
Source: Front Public Health. 2026 Oct 1;14:1966215. doi: 10.3389/fpubh.2026.1966215 (PMC13669201; doi:10.3389/fpubh.2026.1966215)
Supplement: Supplementary file 1 [file Supplementary_file_1.docx]

Supplementary Material

Injury Characteristics and Pre- to Post-Injury Market-Value Change in Professional Football: A Retrospective Cohort Study of 12,948 Injury Episodes

## Supplementary Table S1. Diagnosis dictionary (all Transfermarkt labels in the database)

| **Transfermarkt label** | **Episodes (analysis)** | **Original keyword category** | **Revised category** | **Revised sub-group** | **Coding note** | **Expert review flag** |
| --- | --- | --- | --- | --- | --- | --- |
| Corona virus | 682 | Illness | Illness | COVID-19 | COVID-19 infection | 0 |
| heart problems | 15 | Other | Illness | Cardiovascular | Cardiovascular condition | 0 |
| Circulation problems | 1 | Other | Illness | Cardiovascular | Cardiovascular condition | 0 |
| Vein occlusion | 1 | Other | Illness | Cardiovascular | Cardiovascular condition | 0 |
| Stomach flu | 58 | Other | Illness | Gastrointestinal | GI illness or surgery | 0 |
| stomach problems | 23 | Other | Illness | Gastrointestinal | GI illness or surgery | 0 |
| Appendectomy | 10 | Other | Illness | Gastrointestinal | GI illness or surgery | 0 |
| Intestinal virus | 6 | Illness | Illness | Gastrointestinal | GI illness or surgery | 0 |
| Appendicitis | 5 | Other | Illness | Gastrointestinal | GI illness or surgery | 0 |
| Food poisoning | 2 | Other | Illness | Gastrointestinal | GI illness or surgery | 0 |
| Intestinal surgery | 1 | Other | Illness | Gastrointestinal | GI illness or surgery | 0 |
| Ill | 406 | Illness | Illness | Infection / febrile illness | Infectious or febrile illness | 0 |
| influenza | 95 | Illness | Illness | Infection / febrile illness | Infectious or febrile illness | 0 |
| cold | 86 | Illness | Illness | Infection / febrile illness | Infectious or febrile illness | 0 |
| flu | 82 | Other | Illness | Infection / febrile illness | Infectious or febrile illness | 0 |
| Fever | 66 | Illness | Illness | Infection / febrile illness | Infectious or febrile illness | 0 |
| infection | 57 | Illness | Illness | Infection / febrile illness | Infectious or febrile illness | 0 |
| Virus | 44 | Illness | Illness | Infection / febrile illness | Infectious or febrile illness | 0 |
| Tonsillitis | 29 | Illness | Illness | Infection / febrile illness | Infectious or febrile illness | 0 |
| Mononucleation | 8 | Other | Illness | Infection / febrile illness | Infectious or febrile illness | 0 |
| angina | 6 | Other | Illness | Infection / febrile illness | Infectious or febrile illness | 0 |
| pneumonia | 6 | Other | Illness | Infection / febrile illness | Infectious or febrile illness | 0 |
| bronchitis | 4 | Other | Illness | Infection / febrile illness | Infectious or febrile illness | 0 |
| malaria | 3 | Other | Illness | Infection / febrile illness | Infectious or febrile illness | 0 |
| Tooth infection | 2 | Illness | Illness | Infection / febrile illness | Infectious or febrile illness | 0 |
| Shingles | 1 | Other | Illness | Infection / febrile illness | Infectious or febrile illness | 0 |
| chickenpox | 1 | Other | Illness | Infection / febrile illness | Infectious or febrile illness | 0 |
| depression | 4 | Other | Illness | Mental health | Mental-health condition | 1 |
| Lymphatic cancer | 2 | Other | Illness | Oncological | Oncological condition | 0 |
| Cancer | 1 | Other | Illness | Oncological | Oncological condition | 0 |
| Testicular cancer | 1 | Other | Illness | Oncological | Oncological condition | 0 |
| Dental surgery | 10 | Other | Illness | Other medical | Other medical or dermatological condition | 1 |
| Toothache | 6 | Other | Illness | Other medical | Other medical or dermatological condition | 1 |
| Kidney problems | 2 | Other | Illness | Other medical | Other medical or dermatological condition | 1 |
| Pneumothorax | 2 | Other | Illness | Other medical | Other medical or dermatological condition | 1 |
| Balance disorder | 1 | Other | Illness | Other medical | Other medical or dermatological condition | 1 |
| Inflammation of the pancreas | 1 | Other | Illness | Other medical | Other medical or dermatological condition | 1 |
| allergic reaction | 1 | Other | Illness | Other medical | Other medical or dermatological condition | 1 |
| collapsed lung | 1 | Other | Illness | Other medical | Other medical or dermatological condition | 1 |
| Insect bite | 0 | Other | Illness | Other medical | Other medical or dermatological condition | 1 |
| Skin disease | 0 | Other | Illness | Other medical | Other medical or dermatological condition | 1 |
| Ankle injury | 537 | Joint Injury | Joint/ligament | Ankle (unspecified) | Ankle injury/problem/surgery without tissue detail | 1 |
| Ankle problems | 124 | Joint Injury | Joint/ligament | Ankle (unspecified) | Ankle injury/problem/surgery without tissue detail | 1 |
| Injury to the ankle | 81 | Joint Injury | Joint/ligament | Ankle (unspecified) | Ankle injury/problem/surgery without tissue detail | 1 |
| Ankle surgery | 26 | Joint Injury | Joint/ligament | Ankle (unspecified) | Ankle injury/problem/surgery without tissue detail | 1 |
| Ankle sprain | 101 | Joint Injury | Joint/ligament | Ankle: ligament / sprain | Ankle ligament injury | 0 |
| ankle sprain | 65 | Joint Injury | Joint/ligament | Ankle: ligament / sprain | Ankle ligament injury | 0 |
| Torn ankle ligaments | 29 | Joint Injury | Joint/ligament | Ankle: ligament / sprain | Ankle ligament injury | 0 |
| Torn lateral ankle ligament | 23 | Joint Injury | Joint/ligament | Ankle: ligament / sprain | Ankle ligament injury | 0 |
| Syndesmotic ligament tear | 22 | Joint Injury | Joint/ligament | Ankle: ligament / sprain | Ankle ligament injury | 0 |
| Syndesmosis ligament tear | 16 | Joint Injury | Joint/ligament | Ankle: ligament / sprain | Ankle ligament injury | 0 |
| Ankle ligament tear | 13 | Joint Injury | Joint/ligament | Ankle: ligament / sprain | Ankle ligament injury | 0 |
| Inner ankle ligament tear | 4 | Joint Injury | Joint/ligament | Ankle: ligament / sprain | Ankle ligament injury | 0 |
| Overstretching of the syndesmotic ligament | 2 | Joint Injury | Joint/ligament | Ankle: ligament / sprain | Ankle ligament injury | 0 |
| Capsular tear of ankle joint | 1 | Joint Injury | Joint/ligament | Ankle: ligament / sprain | Ankle ligament injury | 0 |
| Inflammation in the ankle joint | 1 | Joint Injury | Joint/ligament | Ankle: ligament / sprain | Ankle ligament injury | 0 |
| Inner ligament tear in ankle joint | 1 | Joint Injury | Joint/ligament | Ankle: ligament / sprain | Ankle ligament injury | 0 |
| Hip injury | 95 | Joint Injury | Joint/ligament | Hip (unspecified) | Hip injury/problem; v1 keyword 'hip' -> Joint | 1 |
| Hip problems | 49 | Joint Injury | Joint/ligament | Hip (unspecified) | Hip injury/problem; v1 keyword 'hip' -> Joint | 1 |
| Arthroscopy | 3 | Other | Joint/ligament | Joint procedure (unspecified) | Arthroscopy or joint problem without site | 1 |
| Knee injury | 470 | Joint Injury | Joint/ligament | Knee (unspecified) | Knee injury/problem/surgery without tissue detail | 1 |
| Knee problems | 244 | Joint Injury | Joint/ligament | Knee (unspecified) | Knee injury/problem/surgery without tissue detail | 1 |
| Knee surgery | 72 | Joint Injury | Joint/ligament | Knee (unspecified) | Knee injury/problem/surgery without tissue detail | 1 |
| Inflammation in the knee | 17 | Joint Injury | Joint/ligament | Knee (unspecified) | Knee injury/problem/surgery without tissue detail | 1 |
| Edema in the knee | 4 | Joint Injury | Joint/ligament | Knee (unspecified) | Knee injury/problem/surgery without tissue detail | 1 |
| Cyst in the knee | 2 | Joint Injury | Joint/ligament | Knee (unspecified) | Knee injury/problem/surgery without tissue detail | 1 |
| Inner ligament injury | 40 | Joint Injury | Joint/ligament | Knee: collateral ligament | Knee collateral ligament | 0 |
| Inner knee ligament tear | 24 | Joint Injury | Joint/ligament | Knee: collateral ligament | Knee collateral ligament | 0 |
| Torn lateral knee ligament | 19 | Joint Injury | Joint/ligament | Knee: collateral ligament | Knee collateral ligament | 0 |
| Inner ligament stretch of the knee | 17 | Joint Injury | Joint/ligament | Knee: collateral ligament | Knee collateral ligament | 0 |
| Collateral ligament injury | 16 | Joint Injury | Joint/ligament | Knee: collateral ligament | Knee collateral ligament | 0 |
| Knee medial ligament tear | 12 | Joint Injury | Joint/ligament | Knee: collateral ligament | Knee collateral ligament | 0 |
| Outer ligament problems | 11 | Joint Injury | Joint/ligament | Knee: collateral ligament | Knee collateral ligament | 0 |
| Outer ligament tear | 11 | Joint Injury | Joint/ligament | Knee: collateral ligament | Knee collateral ligament | 0 |
| Internal ligament strain | 9 | Joint Injury | Joint/ligament | Knee: collateral ligament | Knee collateral ligament | 0 |
| Internal ligament tear | 8 | Joint Injury | Joint/ligament | Knee: collateral ligament | Knee collateral ligament | 0 |
| Torn knee ligaments | 7 | Joint Injury | Joint/ligament | Knee: collateral ligament | Knee collateral ligament | 0 |
| Knee collateral ligament tear | 4 | Joint Injury | Joint/ligament | Knee: collateral ligament | Knee collateral ligament | 0 |
| Inflammation of ligaments in the knee | 3 | Joint Injury | Joint/ligament | Knee: collateral ligament | Knee collateral ligament | 0 |
| Knee collateral ligament strain | 2 | Joint Injury | Joint/ligament | Knee: collateral ligament | Knee collateral ligament | 0 |
| double ligament tear | 2 | Joint Injury | Joint/ligament | Knee: collateral ligament | Knee collateral ligament | 0 |
| Cruciate ligament tear | 147 | Joint Injury | Joint/ligament | Knee: cruciate ligament | ACL/PCL | 0 |
| Cruciate ligament injury | 23 | Joint Injury | Joint/ligament | Knee: cruciate ligament | ACL/PCL | 0 |
| Cruciate ligament strain | 5 | Joint Injury | Joint/ligament | Knee: cruciate ligament | ACL/PCL | 0 |
| Cruciate ligament surgery | 4 | Joint Injury | Joint/ligament | Knee: cruciate ligament | ACL/PCL | 0 |
| Partial damage to the cruciate ligament | 4 | Joint Injury | Joint/ligament | Knee: cruciate ligament | ACL/PCL | 0 |
| Meniscus injury | 65 | Joint Injury | Joint/ligament | Knee: meniscus / cartilage / patella | Intra-articular knee | 0 |
| Meniscus tear | 24 | Joint Injury | Joint/ligament | Knee: meniscus / cartilage / patella | Intra-articular knee | 0 |
| Meniscus damage | 12 | Joint Injury | Joint/ligament | Knee: meniscus / cartilage / patella | Intra-articular knee | 0 |
| Cartilage damage | 5 | Other | Joint/ligament | Knee: meniscus / cartilage / patella | Intra-articular knee | 0 |
| Tear of the lateral meniscus | 2 | Joint Injury | Joint/ligament | Knee: meniscus / cartilage / patella | Intra-articular knee | 0 |
| Dislocation of the kneecap | 1 | Joint Injury | Joint/ligament | Knee: meniscus / cartilage / patella | Intra-articular knee | 0 |
| Meniscus irritation | 0 | Joint Injury | Joint/ligament | Knee: meniscus / cartilage / patella | Intra-articular knee | 0 |
| Ligament injury | 39 | Joint Injury | Joint/ligament | Ligament / capsule / sprain (other or unspecified) | Ligament or capsular injury without clear site | 1 |
| Capsular injury | 26 | Other | Joint/ligament | Ligament / capsule / sprain (other or unspecified) | Ligament or capsular injury without clear site | 1 |
| Ligament stretching | 11 | Joint Injury | Joint/ligament | Ligament / capsule / sprain (other or unspecified) | Ligament or capsular injury without clear site | 1 |
| sprain | 10 | Other | Joint/ligament | Ligament / capsule / sprain (other or unspecified) | Ligament or capsular injury without clear site | 1 |
| Acromioclavicular joint dislocation | 8 | Other | Joint/ligament | Ligament / capsule / sprain (other or unspecified) | Ligament or capsular injury without clear site | 1 |
| Torn ligaments | 8 | Joint Injury | Joint/ligament | Ligament / capsule / sprain (other or unspecified) | Ligament or capsular injury without clear site | 1 |
| Overstretching | 5 | Other | Joint/ligament | Ligament / capsule / sprain (other or unspecified) | Overstretching = ligament stretch (unspecified) | 1 |
| contortion | 3 | Other | Joint/ligament | Ligament / capsule / sprain (other or unspecified) | Ligament or capsular injury without clear site | 1 |
| Capsule rupture | 2 | Other | Joint/ligament | Ligament / capsule / sprain (other or unspecified) | Ligament or capsular injury without clear site | 1 |
| Ligament tear | 2 | Joint Injury | Joint/ligament | Ligament / capsule / sprain (other or unspecified) | Ligament or capsular injury without clear site | 1 |
| Toe joint capsular tear | 1 | Other | Joint/ligament | Ligament / capsule / sprain (other or unspecified) | Ligament or capsular injury without clear site | 1 |
| Adductor pain | 235 | Muscle Injury | Muscle/tendon | Adductor / groin | Adductor-groin muscle-tendon; 'groin surgery/injury' unspecified | 1 |
| Adductor injury | 158 | Muscle Injury | Muscle/tendon | Adductor / groin | Adductor-groin muscle-tendon; 'groin surgery/injury' unspecified | 1 |
| Groin injury | 154 | Muscle Injury | Muscle/tendon | Adductor / groin | Adductor-groin muscle-tendon; 'groin surgery/injury' unspecified | 1 |
| Groin problems | 57 | Muscle Injury | Muscle/tendon | Adductor / groin | Adductor-groin muscle-tendon; 'groin surgery/injury' unspecified | 1 |
| Groin strain | 38 | Muscle Injury | Muscle/tendon | Adductor / groin | Adductor-groin muscle-tendon; 'groin surgery/injury' unspecified | 1 |
| Groin surgery | 27 | Muscle Injury | Muscle/tendon | Adductor / groin | Adductor-groin muscle-tendon; 'groin surgery/injury' unspecified | 1 |
| Torn muscle fiber in the adductor area | 22 | Muscle Injury | Muscle/tendon | Adductor / groin | Adductor-groin muscle-tendon; 'groin surgery/injury' unspecified | 1 |
| Adductor tear | 7 | Muscle Injury | Muscle/tendon | Adductor / groin | Adductor-groin muscle-tendon; 'groin surgery/injury' unspecified | 1 |
| Calf injury | 262 | Muscle Injury | Muscle/tendon | Calf | Calf muscle | 0 |
| Calf problems | 115 | Muscle Injury | Muscle/tendon | Calf | Calf muscle | 0 |
| Calf strain | 28 | Muscle Injury | Muscle/tendon | Calf | Calf muscle | 0 |
| Calf muscle tear | 18 | Muscle Injury | Muscle/tendon | Calf | Calf muscle | 0 |
| Calf stiffness | 4 | Muscle Injury | Muscle/tendon | Calf | Calf muscle | 0 |
| Hamstring injury | 883 | Muscle Injury | Muscle/tendon | Hamstring | Posterior thigh muscle | 0 |
| Hamstring muscle injury | 123 | Muscle Injury | Muscle/tendon | Hamstring | Posterior thigh muscle | 0 |
| Hamstring strain | 114 | Muscle Injury | Muscle/tendon | Hamstring | Posterior thigh muscle | 0 |
| Abdominal muscle strain | 23 | Muscle Injury | Muscle/tendon | Hip flexor / abdominal | Hip flexor or abdominal muscle; v1 placed 'hip flexor' in Joint (keyword 'hip') | 0 |
| Injury to abdominal muscles | 20 | Muscle Injury | Muscle/tendon | Hip flexor / abdominal | Hip flexor or abdominal muscle; v1 placed 'hip flexor' in Joint (keyword 'hip') | 0 |
| Hip flexor problems | 17 | Joint Injury | Muscle/tendon | Hip flexor / abdominal | Hip flexor or abdominal muscle; v1 placed 'hip flexor' in Joint (keyword 'hip') | 0 |
| Abdominal problems | 9 | Other | Muscle/tendon | Hip flexor / abdominal | Ambiguous: abdominal muscle vs gastrointestinal; kept muscular pending expert review | 1 |
| Right hip flexor problems | 4 | Joint Injury | Muscle/tendon | Hip flexor / abdominal | Hip flexor or abdominal muscle; v1 placed 'hip flexor' in Joint (keyword 'hip') | 0 |
| Left hip flexor problems | 3 | Joint Injury | Muscle/tendon | Hip flexor / abdominal | Hip flexor or abdominal muscle; v1 placed 'hip flexor' in Joint (keyword 'hip') | 0 |
| Achilles tendon problems | 42 | Joint Injury | Muscle/tendon | Tendon (Achilles/patellar/other) | Tendon injury; v1 sent Achilles to Joint and other tendons to Other | 0 |
| Patellar tendon problems | 20 | Other | Muscle/tendon | Tendon (Achilles/patellar/other) | Tendon injury; v1 sent Achilles to Joint and other tendons to Other | 0 |
| Tendon rupture | 15 | Other | Muscle/tendon | Tendon (Achilles/patellar/other) | Tendon injury; v1 sent Achilles to Joint and other tendons to Other | 0 |
| Achilles tendon rupture | 12 | Joint Injury | Muscle/tendon | Tendon (Achilles/patellar/other) | Tendon injury; v1 sent Achilles to Joint and other tendons to Other | 0 |
| Tendon irritation | 12 | Other | Muscle/tendon | Tendon (Achilles/patellar/other) | Tendon injury; v1 sent Achilles to Joint and other tendons to Other | 0 |
| Tendon tear | 9 | Other | Muscle/tendon | Tendon (Achilles/patellar/other) | Tendon injury; v1 sent Achilles to Joint and other tendons to Other | 0 |
| Tendonitis | 8 | Other | Muscle/tendon | Tendon (Achilles/patellar/other) | Tendon injury; v1 sent Achilles to Joint and other tendons to Other | 0 |
| Achilles heel problems | 6 | Joint Injury | Muscle/tendon | Tendon (Achilles/patellar/other) | Tendon injury; v1 sent Achilles to Joint and other tendons to Other | 0 |
| Achilles tendon irritation | 6 | Joint Injury | Muscle/tendon | Tendon (Achilles/patellar/other) | Tendon injury; v1 sent Achilles to Joint and other tendons to Other | 0 |
| Achilles tendon surgery | 6 | Joint Injury | Muscle/tendon | Tendon (Achilles/patellar/other) | Tendon injury; v1 sent Achilles to Joint and other tendons to Other | 0 |
| Patellar tendon irritation | 4 | Other | Muscle/tendon | Tendon (Achilles/patellar/other) | Tendon injury; v1 sent Achilles to Joint and other tendons to Other | 0 |
| Peroneus tendon injury | 3 | Other | Muscle/tendon | Tendon (Achilles/patellar/other) | Tendon injury; v1 sent Achilles to Joint and other tendons to Other | 0 |
| Patellar tendinopathy syndrome | 2 | Other | Muscle/tendon | Tendon (Achilles/patellar/other) | Tendon injury; v1 sent Achilles to Joint and other tendons to Other | 0 |
| Patellar tendon dislocation | 2 | Other | Muscle/tendon | Tendon (Achilles/patellar/other) | Tendon injury; v1 sent Achilles to Joint and other tendons to Other | 0 |
| Patellar tendon rupture | 2 | Other | Muscle/tendon | Tendon (Achilles/patellar/other) | Tendon injury; v1 sent Achilles to Joint and other tendons to Other | 0 |
| Patellar tendon tear | 1 | Other | Muscle/tendon | Tendon (Achilles/patellar/other) | Tendon injury; v1 sent Achilles to Joint and other tendons to Other | 0 |
| Inflammation of the biceps tendon in the thigh | 0 | Muscle Injury | Muscle/tendon | Tendon (Achilles/patellar/other) | Tendon injury; v1 sent Achilles to Joint and other tendons to Other | 0 |
| Partial patellar tendon tear | 0 | Other | Muscle/tendon | Tendon (Achilles/patellar/other) | Tendon injury; v1 sent Achilles to Joint and other tendons to Other | 0 |
| Thigh problems | 271 | Muscle Injury | Muscle/tendon | Thigh / quadriceps / gluteal | Anterior thigh or gluteal muscle | 0 |
| Torn thigh muscle | 24 | Muscle Injury | Muscle/tendon | Thigh / quadriceps / gluteal | Anterior thigh or gluteal muscle | 0 |
| Strain in the thigh and gluteal muscles | 18 | Muscle Injury | Muscle/tendon | Thigh / quadriceps / gluteal | Anterior thigh or gluteal muscle | 0 |
| Muscle injury | 751 | Muscle Injury | Muscle/tendon | Unspecified muscle | Muscle injury without site; bare 'strain' assumed muscular | 1 |
| muscular problems | 606 | Muscle Injury | Muscle/tendon | Unspecified muscle | Muscle injury without site; bare 'strain' assumed muscular | 1 |
| Torn muscle fiber | 207 | Muscle Injury | Muscle/tendon | Unspecified muscle | Muscle injury without site; bare 'strain' assumed muscular | 1 |
| Muscle fatigue | 157 | Muscle Injury | Muscle/tendon | Unspecified muscle | Muscle injury without site; bare 'strain' assumed muscular | 1 |
| Torn muscle bundle | 53 | Muscle Injury | Muscle/tendon | Unspecified muscle | Muscle injury without site; bare 'strain' assumed muscular | 1 |
| strain | 39 | Other | Muscle/tendon | Unspecified muscle | Muscle injury without site; bare 'strain' assumed muscular | 1 |
| Muscle strain | 36 | Muscle Injury | Muscle/tendon | Unspecified muscle | Muscle injury without site; bare 'strain' assumed muscular | 1 |
| Muscle tear | 21 | Muscle Injury | Muscle/tendon | Unspecified muscle | Muscle injury without site; bare 'strain' assumed muscular | 1 |
| Contracture | 20 | Other | Muscle/tendon | Unspecified muscle | Muscle injury without site; bare 'strain' assumed muscular | 1 |
| muscle stiffness | 11 | Muscle Injury | Muscle/tendon | Unspecified muscle | Muscle injury without site; bare 'strain' assumed muscular | 1 |
| Muscle fiber tear | 9 | Muscle Injury | Muscle/tendon | Unspecified muscle | Muscle injury without site; bare 'strain' assumed muscular | 1 |
| Partial muscle tear | 2 | Muscle Injury | Muscle/tendon | Unspecified muscle | Muscle injury without site; bare 'strain' assumed muscular | 1 |
| Sore muscles | 2 | Muscle Injury | Muscle/tendon | Unspecified muscle | Muscle injury without site; bare 'strain' assumed muscular | 1 |
| Knock | 398 | Other | Other | Contusion / knock | Direct-impact contusion incl. 'dead leg' (thigh contusion) | 0 |
| minor knock | 138 | Other | Other | Contusion / knock | Direct-impact contusion incl. 'dead leg' (thigh contusion) | 0 |
| Dead leg | 110 | Other | Other | Contusion / knock | Direct-impact contusion incl. 'dead leg' (thigh contusion) | 0 |
| bruise | 101 | Other | Other | Contusion / knock | Direct-impact contusion incl. 'dead leg' (thigh contusion) | 0 |
| Knee bruise | 34 | Joint Injury | Other | Contusion / knock | Direct-impact contusion incl. 'dead leg' (thigh contusion) | 0 |
| Foot bruise | 33 | Other | Other | Contusion / knock | Direct-impact contusion incl. 'dead leg' (thigh contusion) | 0 |
| Bruised ribs | 24 | Other | Other | Contusion / knock | Direct-impact contusion incl. 'dead leg' (thigh contusion) | 0 |
| Hip bruise | 15 | Joint Injury | Other | Contusion / knock | Direct-impact contusion incl. 'dead leg' (thigh contusion) | 0 |
| Bruise on ankle | 14 | Joint Injury | Other | Contusion / knock | Direct-impact contusion incl. 'dead leg' (thigh contusion) | 0 |
| Bruised knee | 11 | Joint Injury | Other | Contusion / knock | Direct-impact contusion incl. 'dead leg' (thigh contusion) | 0 |
| Shin bruise | 11 | Other | Other | Contusion / knock | Direct-impact contusion incl. 'dead leg' (thigh contusion) | 0 |
| Muscle contusion | 10 | Muscle Injury | Other | Contusion / knock | Direct-impact contusion incl. 'dead leg' (thigh contusion) | 0 |
| Achilles tendon contusion | 8 | Joint Injury | Other | Contusion / knock | Direct-impact contusion incl. 'dead leg' (thigh contusion) | 0 |
| Metatarsal bruise | 8 | Other | Other | Contusion / knock | Direct-impact contusion incl. 'dead leg' (thigh contusion) | 0 |
| Pelvic contusion | 6 | Other | Other | Contusion / knock | Direct-impact contusion incl. 'dead leg' (thigh contusion) | 0 |
| Bruise on the ankle joint | 5 | Joint Injury | Other | Contusion / knock | Direct-impact contusion incl. 'dead leg' (thigh contusion) | 0 |
| Bruised back | 4 | Other | Other | Contusion / knock | Direct-impact contusion incl. 'dead leg' (thigh contusion) | 0 |
| Shoulder joint contusion | 2 | Other | Other | Contusion / knock | Direct-impact contusion incl. 'dead leg' (thigh contusion) | 0 |
| Bruise on shinbone | 1 | Other | Other | Contusion / knock | Direct-impact contusion incl. 'dead leg' (thigh contusion) | 0 |
| Cheekbone bruise | 1 | Other | Other | Contusion / knock | Direct-impact contusion incl. 'dead leg' (thigh contusion) | 0 |
| Neck bruise | 1 | Other | Other | Contusion / knock | Direct-impact contusion incl. 'dead leg' (thigh contusion) | 0 |
| Metatarsal fracture | 67 | Other | Other | Fracture / bone stress | Bone injury (incl. facial and spinal fractures) | 0 |
| Broken nose bone | 26 | Other | Other | Fracture / bone stress | Bone injury (incl. facial and spinal fractures) | 0 |
| Broken collarbone | 23 | Other | Other | Fracture / bone stress | Bone injury (incl. facial and spinal fractures) | 0 |
| Broken fibula | 22 | Other | Other | Fracture / bone stress | Bone injury (incl. facial and spinal fractures) | 0 |
| Broken toe | 22 | Other | Other | Fracture / bone stress | Bone injury (incl. facial and spinal fractures) | 0 |
| Rib fracture | 17 | Other | Other | Fracture / bone stress | Bone injury (incl. facial and spinal fractures) | 0 |
| Broken foot | 16 | Other | Other | Fracture / bone stress | Bone injury (incl. facial and spinal fractures) | 0 |
| Bone edema | 15 | Other | Other | Fracture / bone stress | Bone injury (incl. facial and spinal fractures) | 0 |
| Broken ankle | 14 | Joint Injury | Other | Fracture / bone stress | Bone injury (incl. facial and spinal fractures) | 0 |
| Metacarpal fracture | 14 | Other | Other | Fracture / bone stress | Bone injury (incl. facial and spinal fractures) | 0 |
| Broken cheekbone | 12 | Other | Other | Fracture / bone stress | Bone injury (incl. facial and spinal fractures) | 0 |
| Broken finger | 10 | Other | Other | Fracture / bone stress | Bone injury (incl. facial and spinal fractures) | 0 |
| Facial fracture | 10 | Other | Other | Fracture / bone stress | Bone injury (incl. facial and spinal fractures) | 0 |
| Scaphoid fracture | 9 | Other | Other | Fracture / bone stress | Bone injury (incl. facial and spinal fractures) | 0 |
| Stress reaction of the bone | 9 | Other | Other | Fracture / bone stress | Bone injury (incl. facial and spinal fractures) | 0 |
| Broken arm | 8 | Other | Other | Fracture / bone stress | Bone injury (incl. facial and spinal fractures) | 0 |
| Broken hand | 8 | Other | Other | Fracture / bone stress | Bone injury (incl. facial and spinal fractures) | 0 |
| Wrist fracture | 7 | Other | Other | Fracture / bone stress | Bone injury (incl. facial and spinal fractures) | 0 |
| fatigue fracture | 7 | Other | Other | Fracture / bone stress | Bone injury (incl. facial and spinal fractures) | 0 |
| Lumbar vertebra fracture | 6 | Other | Other | Fracture / bone stress | Bone injury (incl. facial and spinal fractures) | 0 |
| Broken jaw | 5 | Other | Other | Fracture / bone stress | Bone injury (incl. facial and spinal fractures) | 0 |
| Hairline crack in foot | 5 | Other | Other | Fracture / bone stress | Bone injury (incl. facial and spinal fractures) | 0 |
| Broken tibia | 4 | Other | Other | Fracture / bone stress | Bone injury (incl. facial and spinal fractures) | 0 |
| Eyebow fracture | 4 | Other | Other | Fracture / bone stress | Bone injury (incl. facial and spinal fractures) | 0 |
| Forearm fracture | 4 | Other | Other | Fracture / bone stress | Bone injury (incl. facial and spinal fractures) | 0 |
| fracture | 4 | Other | Other | Fracture / bone stress | Bone injury (incl. facial and spinal fractures) | 0 |
| Bone bruise | 3 | Other | Other | Fracture / bone stress | Bone injury (incl. facial and spinal fractures) | 0 |
| Bone sprain | 3 | Other | Other | Fracture / bone stress | Bone injury (incl. facial and spinal fractures) | 0 |
| Broken leg | 3 | Other | Other | Fracture / bone stress | Bone injury (incl. facial and spinal fractures) | 0 |
| Elbow fracture | 3 | Other | Other | Fracture / bone stress | Bone injury (incl. facial and spinal fractures) | 0 |
| Fracture of the eye socket | 3 | Other | Other | Fracture / bone stress | Bone injury (incl. facial and spinal fractures) | 0 |
| Hairline fracture in the fibula | 3 | Other | Other | Fracture / bone stress | Bone injury (incl. facial and spinal fractures) | 0 |
| Broken shoulder | 2 | Other | Other | Fracture / bone stress | Bone injury (incl. facial and spinal fractures) | 0 |
| Cervical vertebra fracture | 2 | Other | Other | Fracture / bone stress | Bone injury (incl. facial and spinal fractures) | 0 |
| Dislocation fracture of the ankle joint | 2 | Joint Injury | Other | Fracture / bone stress | Bone injury (incl. facial and spinal fractures) | 0 |
| Fissure of the fibula | 2 | Other | Other | Fracture / bone stress | Bone injury (incl. facial and spinal fractures) | 0 |
| Fracture of fibula shaft | 2 | Other | Other | Fracture / bone stress | Bone injury (incl. facial and spinal fractures) | 0 |
| Hairline crack in middle finger | 2 | Other | Other | Fracture / bone stress | Bone injury (incl. facial and spinal fractures) | 0 |
| Lower leg fracture | 2 | Other | Other | Fracture / bone stress | Bone injury (incl. facial and spinal fractures) | 0 |
| Skull fracture | 2 | Other | Other | Fracture / bone stress | Bone injury (incl. facial and spinal fractures) | 0 |
| Broken thumb | 1 | Other | Other | Fracture / bone stress | Bone injury (incl. facial and spinal fractures) | 0 |
| Crack bruise | 1 | Other | Other | Fracture / bone stress | Bone injury (incl. facial and spinal fractures) | 0 |
| Femoral neck fracture | 1 | Other | Other | Fracture / bone stress | Bone injury (incl. facial and spinal fractures) | 0 |
| Fracture of frontal bone | 1 | Other | Other | Fracture / bone stress | Bone injury (incl. facial and spinal fractures) | 0 |
| Hairline fracture in the muscles | 1 | Muscle Injury | Other | Fracture / bone stress | Bone injury (incl. facial and spinal fractures) | 0 |
| Broken kneecap | 0 | Joint Injury | Other | Fracture / bone stress | Bone injury (incl. facial and spinal fractures) | 0 |
| Pubalgia | 33 | Other | Other | Groin-pubic overuse / hernia | Pubic-groin overuse (osteitis pubis, pubalgia) or inguinal hernia; not a muscle strain; v1 placed in Other | 1 |
| Inflammation of pubic bone | 23 | Other | Other | Groin-pubic overuse / hernia | Pubic-groin overuse (osteitis pubis, pubalgia) or inguinal hernia; not a muscle strain; v1 placed in Other | 1 |
| Pubic bone irritation | 12 | Other | Other | Groin-pubic overuse / hernia | Pubic-groin overuse (osteitis pubis, pubalgia) or inguinal hernia; not a muscle strain; v1 placed in Other | 1 |
| Inguinal hernia | 6 | Other | Other | Groin-pubic overuse / hernia | Pubic-groin overuse (osteitis pubis, pubalgia) or inguinal hernia; not a muscle strain; v1 placed in Other | 1 |
| Herniated disc | 5 | Other | Other | Groin-pubic overuse / hernia | Pubic-groin overuse (osteitis pubis, pubalgia) or inguinal hernia; not a muscle strain; v1 placed in Other | 1 |
| Pubic bone bruise | 2 | Other | Other | Groin-pubic overuse / hernia | Pubic-groin overuse (osteitis pubis, pubalgia) or inguinal hernia; not a muscle strain; v1 placed in Other | 1 |
| Head injury | 71 | Other | Other | Head, face or neck | Head/face/neck injury (concussion = CNS) | 0 |
| concussion | 56 | Other | Other | Head, face or neck | Head/face/neck injury (concussion = CNS) | 0 |
| Eye injury | 16 | Other | Other | Head, face or neck | Head/face/neck injury (concussion = CNS) | 0 |
| Neck injury | 9 | Other | Other | Head, face or neck | Head/face/neck injury (concussion = CNS) | 0 |
| Nose injury | 9 | Other | Other | Head, face or neck | Head/face/neck injury (concussion = CNS) | 0 |
| Facial injury | 8 | Other | Other | Head, face or neck | Head/face/neck injury (concussion = CNS) | 0 |
| Nose surgery | 8 | Other | Other | Head, face or neck | Head/face/neck injury (concussion = CNS) | 0 |
| Whiplash | 3 | Joint Injury | Other | Head, face or neck | Head/face/neck injury (concussion = CNS) | 0 |
| Cyst on jaw | 1 | Other | Other | Head, face or neck | Head/face/neck injury (concussion = CNS) | 0 |
| Ruptured eardrum | 1 | Other | Other | Head, face or neck | Head/face/neck injury (concussion = CNS) | 0 |
| flesh wound | 8 | Other | Other | Laceration / wound | Skin laceration | 0 |
| Cut | 7 | Other | Other | Laceration / wound | Skin laceration | 0 |
| laceration wound | 5 | Other | Other | Laceration / wound | Skin laceration | 0 |
| laceration | 4 | Other | Other | Laceration / wound | Skin laceration | 0 |
| open wound | 1 | Other | Other | Laceration / wound | Skin laceration | 0 |
| inflamed wound | 0 | Other | Other | Laceration / wound | Skin laceration | 0 |
| Fitness | 248 | Other | Other | Non-clinical absence (fitness/rest) | Not an injury; retained in Other as in v1 | 1 |
| Rest | 115 | Other | Other | Non-clinical absence (fitness/rest) | Not an injury; retained in Other as in v1 | 1 |
| Quarantine | 121 | Other | Other | Non-clinical absence (quarantine) | COVID-era isolation without documented illness; expert may reassign to Illness | 1 |
| inflammation | 18 | Other | Other | Overuse / inflammation (unspecified) | Overuse or inflammatory condition without tissue detail | 1 |
| Arch problems | 7 | Other | Other | Overuse / inflammation (unspecified) | Overuse or inflammatory condition without tissue detail | 1 |
| Heel spur | 5 | Other | Other | Overuse / inflammation (unspecified) | Overuse or inflammatory condition without tissue detail | 1 |
| Inflammation of the sole of the foot | 4 | Other | Other | Overuse / inflammation (unspecified) | Overuse or inflammatory condition without tissue detail | 1 |
| Bursitis | 3 | Other | Other | Overuse / inflammation (unspecified) | Overuse or inflammatory condition without tissue detail | 1 |
| Inflammation in the head of the fibula | 1 | Other | Other | Overuse / inflammation (unspecified) | Overuse or inflammatory condition without tissue detail | 1 |
| Bone inflammation | 0 | Other | Other | Overuse / inflammation (unspecified) | Overuse or inflammatory condition without tissue detail | 1 |
| Back problems | 94 | Other | Other | Spine / back | Spinal or back condition | 0 |
| Back injury | 83 | Other | Other | Spine / back | Spinal or back condition | 0 |
| Lumbago | 36 | Other | Other | Spine / back | Spinal or back condition | 0 |
| Sciatica problems | 3 | Other | Other | Spine / back | Spinal or back condition | 0 |
| Vertebral injury | 3 | Other | Other | Spine / back | Spinal or back condition | 0 |
| Cervical spine injury | 2 | Other | Other | Spine / back | Spinal or back condition | 0 |
| Inflammation in the spine | 2 | Other | Other | Spine / back | Spinal or back condition | 0 |
| Compression of the spine | 1 | Other | Other | Spine / back | Spinal or back condition | 0 |
| Lumbar vertebra problems | 1 | Other | Other | Spine / back | Spinal or back condition | 0 |
| pinched nerve | 1 | Other | Other | Spine / back | Spinal or back condition | 0 |
| Blockage in the back | 0 | Other | Other | Spine / back | Spinal or back condition | 0 |
| surgery | 41 | Other | Other | Surgery / procedure (unspecified) | Unspecified surgery | 1 |
| Remove screws/nails | 2 | Other | Other | Surgery / procedure (unspecified) | Hardware removal after fracture | 0 |
| unknown injury | 885 | Other | Other | Unknown injury | Transfermarkt 'unknown injury' | 0 |
| traffic accident | 2 | Other | Other | Unspecified injury (accident) | Accident, injury unspecified | 1 |
| Shoulder injury | 225 | Other | Other | Unspecified injury by body region | Region given, tissue unspecified (v1 also Other) | 1 |
| Foot injury | 155 | Other | Other | Unspecified injury by body region | Region given, tissue unspecified (v1 also Other) | 1 |
| Leg injury | 47 | Other | Other | Unspecified injury by body region | Region given, tissue unspecified (v1 also Other) | 1 |
| Toe injury | 35 | Other | Other | Unspecified injury by body region | Region given, tissue unspecified (v1 also Other) | 1 |
| Hand injury | 29 | Other | Other | Unspecified injury by body region | Region given, tissue unspecified (v1 also Other) | 1 |
| Elbow injury | 22 | Other | Other | Unspecified injury by body region | Region given, tissue unspecified (v1 also Other) | 1 |
| Finger injury | 20 | Other | Other | Unspecified injury by body region | Region given, tissue unspecified (v1 also Other) | 1 |
| Shin injury | 17 | Other | Other | Unspecified injury by body region | Region given, tissue unspecified (v1 also Other) | 1 |
| Arm injury | 15 | Other | Other | Unspecified injury by body region | Region given, tissue unspecified (v1 also Other) | 1 |
| Heel injury | 15 | Other | Other | Unspecified injury by body region | Region given, tissue unspecified (v1 also Other) | 1 |
| Heel problems | 12 | Other | Other | Unspecified injury by body region | Region given, tissue unspecified (v1 also Other) | 1 |
| Foot surgery | 11 | Other | Other | Unspecified injury by body region | Region given, tissue unspecified (v1 also Other) | 1 |
| Wrist injury | 11 | Other | Other | Unspecified injury by body region | Region given, tissue unspecified (v1 also Other) | 1 |
| Chest injury | 10 | Other | Other | Unspecified injury by body region | Region given, tissue unspecified (v1 also Other) | 1 |
| Pelvic injury | 7 | Other | Other | Unspecified injury by body region | Region given, tissue unspecified (v1 also Other) | 1 |
| Thumb injury | 3 | Other | Other | Unspecified injury by body region | Region given, tissue unspecified (v1 also Other) | 1 |
| Scaphoid surgery | 1 | Other | Other | Unspecified injury by body region | Region given, tissue unspecified (v1 also Other) | 1 |
| Testicle rupture | 1 | Other | Other | Unspecified injury by body region | Region given, tissue unspecified (v1 also Other) | 1 |

Revised categories follow the tissue-type classification of Fuller et al. (2006). Expert review flag = 1 where the tissue cannot be determined from the label alone.

## Supplementary Table S2. Composition of the 'other' category (revised dictionary)

| **Sub-group** | **Episodes** | **Players** | **Mean** | **SD** | **Median** | **Q1** | **Q3** | **Mean duration** | **Median duration** |
| --- | --- | --- | --- | --- | --- | --- | --- | --- | --- |
| Contusion / knock | 935 | 578 | 76.6 | 215.2 | 10.0 | -20.0 | 80.0 | 16.3 | 10.0 |
| Unknown injury | 885 | 525 | 59.0 | 178.5 | 0.0 | -25.0 | 66.7 | 21.4 | 13.0 |
| Unspecified injury by body region | 636 | 491 | 79.5 | 230.5 | 0.0 | -20.0 | 87.5 | 41.8 | 24.5 |
| Fracture / bone stress | 387 | 336 | 85.6 | 215.7 | 11.1 | -20.0 | 100.0 | 59.0 | 45.0 |
| Non-clinical absence (fitness/rest) | 363 | 286 | 41.9 | 142.2 | 0.0 | -25.0 | 42.9 | 16.7 | 8.0 |
| Spine / back | 226 | 176 | 51.1 | 208.2 | 0.0 | -27.1 | 33.3 | 29.7 | 13.0 |
| Head, face or neck | 182 | 164 | 91.0 | 242.1 | 7.3 | -25.0 | 100.0 | 14.6 | 9.0 |
| Non-clinical absence (quarantine) | 121 | 111 | 48.0 | 124.5 | 20.0 | -15.0 | 75.0 | 10.0 | 8.0 |
| Groin-pubic overuse / hernia | 81 | 71 | 82.4 | 216.8 | 0.0 | -25.0 | 71.4 | 53.3 | 38.0 |
| Surgery / procedure (unspecified) | 43 | 41 | 87.2 | 212.7 | 0.0 | -18.9 | 110.0 | 69.8 | 57.0 |
| Overuse / inflammation (unspecified) | 38 | 37 | 45.2 | 117.2 | 2.6 | -14.8 | 44.7 | 26.3 | 12.0 |
| Laceration / wound | 25 | 24 | 148.8 | 258.3 | 75.0 | 16.7 | 113.3 | 13.1 | 9.0 |
| Unspecified injury (accident) | 2 | 2 | -37.5 | 17.7 | -37.5 | -43.8 | -31.2 | 4.5 | 4.5 |

## Supplementary Table S3. Originally submitted Models 1–3 with conventional and player-clustered standard errors

| **Model (as submitted)** | **Term** | **B** | **SE (conventional)** | **p (conventional)** | **SE (player-clustered)** | **p (clustered)** | **SE ratio** |
| --- | --- | --- | --- | --- | --- | --- | --- |
| M1_original | Intercept | 529.9596 | 11.8313 | 0.0 | 18.8606 | 0.0 | 1.5941 |
| M1_original | C(injury_category_v1)[T.Joint Injury] | -8.4865 | 6.3134 | 0.1789 | 6.8422 | 0.215 | 1.0838 |
| M1_original | C(injury_category_v1)[T.Muscle Injury] | -9.3326 | 5.7041 | 0.1018 | 6.134 | 0.1283 | 1.0754 |
| M1_original | C(injury_category_v1)[T.Other] | -6.8071 | 5.73 | 0.2349 | 6.2455 | 0.2759 | 1.09 |
| M1_original | duration_days | -0.1444 | 0.0388 | 0.0002 | 0.0415 | 0.0005 | 1.0691 |
| M1_original | age_original | -18.7341 | 0.4436 | 0.0 | 0.679 | 0.0 | 1.5307 |
| M2_original | Intercept | 543.3773 | 12.2461 | 0.0 | 19.5519 | 0.0 | 1.5966 |
| M2_original | C(injury_category_v1)[T.Joint Injury] | -7.3231 | 6.31 | 0.2458 | 6.8501 | 0.2852 | 1.0856 |
| M2_original | C(injury_category_v1)[T.Muscle Injury] | -7.3418 | 5.7144 | 0.1989 | 6.1634 | 0.2337 | 1.0786 |
| M2_original | C(injury_category_v1)[T.Other] | -6.7854 | 5.7245 | 0.2359 | 6.2554 | 0.2782 | 1.0927 |
| M2_original | C(position_group)[T.Forward] | -11.054 | 4.2056 | 0.0086 | 5.6845 | 0.052 | 1.3516 |
| M2_original | C(position_group)[T.Goalkeeper] | 24.3079 | 6.4589 | 0.0002 | 7.4441 | 0.0011 | 1.1525 |
| M2_original | C(position_group)[T.Midfielder] | -9.6757 | 4.1552 | 0.0199 | 5.3761 | 0.072 | 1.2938 |
| M2_original | C(position_group)[T.Unknown] | -77.77 | 54.3629 | 0.1526 | 4.0356 | 0.0 | 0.0742 |
| M2_original | duration_days | -0.1546 | 0.0388 | 0.0001 | 0.0416 | 0.0002 | 1.0733 |
| M2_original | age_original | -19.1572 | 0.4491 | 0.0 | 0.6918 | 0.0 | 1.5405 |
| M3_original | Intercept | 545.1368 | 11.5911 | 0.0 | 18.5197 | 0.0 | 1.5978 |
| M3_original | C(injury_category_v1)[T.Joint Injury] | -6.4515 | 6.1766 | 0.2963 | 6.6707 | 0.3336 | 1.08 |
| M3_original | C(injury_category_v1)[T.Muscle Injury] | -3.9906 | 5.5844 | 0.4749 | 5.9958 | 0.5058 | 1.0737 |
| M3_original | C(injury_category_v1)[T.Other] | -8.7782 | 5.606 | 0.1174 | 6.0972 | 0.1501 | 1.0876 |
| M3_original | duration_days | -0.2 | 0.038 | 0.0 | 0.0407 | 0.0 | 1.071 |
| M3_original | age_original | -18.1714 | 0.4345 | 0.0 | 0.6428 | 0.0 | 1.4792 |
| M3_original | baseline_m | -2.02 | 0.0837 | 0.0 | 0.1653 | 0.0 | 1.9754 |

Outcome = rounded percentage change as stored; age = valuation-record age; injury category = original keyword classification (reference: illness). Coefficients are unchanged by the covariance estimator.

## Supplementary Table S3b. Models 1–4: all coefficients including position, season and league group

| **Term** | **Model 1** | **Model 2** | **Model 3** | **Model 4** |
| --- | --- | --- | --- | --- |
| Intercept | 57.0 (50.6 to 63.4); <0.001 | 65.1 (54.0 to 76.1); <0.001 | 91.4 (83.0 to 99.7); <0.001 | 114.4 (95.8 to 133.0); <0.001 |
| Injury category: Illness | 6.4 (-4.7 to 17.4); 0.259 | 4.1 (-7.0 to 15.2); 0.468 | 1.3 (-9.5 to 12.1); 0.816 | 1.0 (-10.4 to 12.3); 0.865 |
| Injury category: Joint/ligament | 1.2 (-8.0 to 10.4); 0.793 | 0.3 (-8.9 to 9.5); 0.957 | -2.3 (-11.3 to 6.8); 0.622 | -5.4 (-14.4 to 3.5); 0.231 |
| Injury category: Other | 2.4 (-5.7 to 10.5); 0.564 | 0.3 (-7.8 to 8.5); 0.942 | -5.1 (-13.1 to 3.0); 0.216 | -8.9 (-17.1 to -0.7); 0.034 |
| Injury duration (per 30 days) | -4.5 (-6.9 to -2.0); <0.001 | -4.8 (-7.3 to -2.4); <0.001 | -6.1 (-8.5 to -3.8); <0.001 | — |
| Age (per year, centred at 25) | -18.8 (-20.2 to -17.5); <0.001 | -19.4 (-20.7 to -18.0); <0.001 | -18.3 (-19.6 to -17.0); <0.001 | -18.2 (-19.7 to -16.8); <0.001 |
| Position: Second Striker | — | -35.1 (-64.9 to -5.4); 0.021 | — | -17.4 (-50.7 to 15.9); 0.305 |
| Position: Right-Back | — | -4.7 (-20.0 to 10.6); 0.545 | — | -12.2 (-27.0 to 2.6); 0.106 |
| Position: Right Winger | — | -19.9 (-41.3 to 1.5); 0.068 | — | -9.0 (-30.6 to 12.6); 0.413 |
| Position: Right Midfield | — | -3.6 (-35.7 to 28.4); 0.823 | — | -8.2 (-37.5 to 21.0); 0.581 |
| Position: Left-Back | — | -8.4 (-26.5 to 9.7); 0.362 | — | -12.4 (-30.3 to 5.4); 0.173 |
| Position: Left Winger | — | -17.8 (-38.0 to 2.5); 0.085 | — | -9.1 (-29.3 to 11.1); 0.377 |
| Position: Left Midfield | — | -40.1 (-77.9 to -2.2); 0.038 | — | -43.6 (-78.4 to -8.7); 0.014 |
| Position: Defensive Midfield | — | -8.5 (-25.6 to 8.5); 0.328 | — | -9.2 (-25.5 to 7.0); 0.266 |
| Position: Centre-Forward | — | -8.1 (-23.8 to 7.6); 0.313 | — | -1.4 (-17.0 to 14.1); 0.859 |
| Position: Central Midfield | — | -14.6 (-29.8 to 0.6); 0.060 | — | -13.0 (-28.3 to 2.2); 0.094 |
| Position: Attacking Midfield | — | -11.5 (-33.4 to 10.4); 0.305 | — | 1.0 (-20.2 to 22.3); 0.925 |
| Position: Goalkeeper | — | 22.0 (5.9 to 38.0); 0.007 | — | 14.3 (-2.9 to 31.5); 0.103 |
| Pre-injury market value (per EUR 1 million) | — | — | -2.0 (-2.3 to -1.7); <0.001 | -1.9 (-2.3 to -1.6); <0.001 |
| Season: 13/14 | — | — | — | -38.5 (-76.6 to -0.4); 0.048 |
| Season: 14/15 | — | — | — | -39.7 (-74.4 to -5.1); 0.025 |
| Season: 15/16 | — | — | — | 8.7 (-32.8 to 50.3); 0.681 |
| Season: 16/17 | — | — | — | 7.3 (-23.2 to 37.7); 0.640 |
| Season: 17/18 | — | — | — | 37.4 (10.3 to 64.5); 0.007 |
| Season: 18/19 | — | — | — | 32.4 (11.1 to 53.7); 0.003 |
| Season: 19/20 | — | — | — | -30.0 (-43.2 to -16.8); <0.001 |
| Season: 20/21 | — | — | — | -11.2 (-23.8 to 1.4); 0.082 |
| Season: 21/22 | — | — | — | -19.9 (-32.9 to -7.0); 0.003 |
| Season: 24/25 | — | — | — | -17.9 (-46.8 to 11.0); 0.224 |
| League group: ES1 | — | — | — | -1.1 (-13.9 to 11.7); 0.864 |
| League group: FR1 | — | — | — | -4.7 (-21.6 to 12.1); 0.583 |
| League group: IT1 | — | — | — | -11.0 (-22.6 to 0.7); 0.065 |
| League group: L1 | — | — | — | -20.3 (-32.3 to -8.4); <0.001 |
| League group: Other top division | — | — | — | 11.6 (-6.8 to 30.0); 0.217 |
| League group: Other/cup/youth/regional | — | — | — | -30.8 (-54.7 to -7.0); 0.011 |
| League group: Second tier | — | — | — | 7.8 (-13.7 to 29.3); 0.477 |
| League group: Unknown | — | — | — | 34.4 (-14.8 to 83.6); 0.170 |
| Season: 12/13 | — | — | — | -27.7 (-87.8 to 32.5); 0.367 |
| Season: 22/23 | — | — | — | -19.5 (-30.3 to -8.7); <0.001 |
| Season: 11/12 | — | — | — | -76.8 (-115.2 to -38.4); <0.001 |
| Season: 09/10 | — | — | — | 15.4 (-122.6 to 153.3); 0.827 |
| Duration group: 15-30 | — | — | — | -11.4 (-19.6 to -3.2); 0.006 |
| Duration group: 31-90 | — | — | — | -23.6 (-32.4 to -14.8); <0.001 |
| Duration group: >90 | — | — | — | -26.4 (-42.6 to -10.1); 0.002 |
| Season: 10/11 | — | — | — | -82.5 (-127.3 to -37.6); <0.001 |
| Season: 08/09 | — | — | — | -68.3 (-164.9 to 28.2); 0.165 |
| Episodes / players | 12,936 / 2,059 | 12,936 / 2,059 | 12,936 / 2,059 | 12,936 / 2,059 |
| R² / adjusted R² | 0.1241 / 0.1238 | 0.1271 / 0.1260 | 0.1619 / 0.1615 | 0.1772 / 0.1744 |

Note. All models were fitted on the same 12,936 episodes from 2,059 players; standard errors are clustered by player (CR1) with t-based confidence intervals. Reference categories: muscle/tendon injury; 1–14 days; centre-back; season 2023/24; English top division. Age is centred at 25 years; injury duration is per 30 days in Models 1–3 and in groups in Model 4; pre-injury value is per EUR 1 million. Coefficients for playing position, season and league group are given in Supplementary Table S3b.

## Supplementary Table S4. Kruskal–Wallis tests with effect sizes (all episodes and earliest episode per player)

| **Sample** | **Variable** | **H** | **df** | **n** | **Players** | **p** | **η²H** |
| --- | --- | --- | --- | --- | --- | --- | --- |
| all events (clustered within players; exploratory) | injury_category (v2) | 34.3504 | 3 | 12948 | 2061 | 0.0 | 0.0024 |
| all events (clustered within players; exploratory) | duration_group | 36.0062 | 3 | 12948 | 2061 | 0.0 | 0.0025 |
| all events (clustered within players; exploratory) | age_group (exact age) | 2873.0653 | 3 | 12936 | 2059 | 0.0 | 0.2219 |
| all events (clustered within players; exploratory) | age_group (original age variable) | 2830.0199 | 3 | 12948 | 2061 | 0.0 | 0.2184 |
| all events (clustered within players; exploratory) | injury_category (v1, original keywords) | 30.2989 | 3 | 12948 | 2061 | 0.0 | 0.0021 |
| all events (clustered within players; exploratory) | position_group | 7.5665 | 4 | 12948 | 2061 | 0.1088 | 0.0003 |
| first event per player (independent observations) | injury_category (v2) | 2.7187 | 3 | 2061 | 2061 | 0.4371 | -0.0001 |
| first event per player (independent observations) | duration_group | 13.7876 | 3 | 2061 | 2061 | 0.0032 | 0.0052 |
| first event per player (independent observations) | age_group (exact age) | 255.939 | 3 | 2059 | 2059 | 0.0 | 0.1231 |
| first event per player (independent observations) | age_group (original age variable) | 253.3697 | 3 | 2061 | 2061 | 0.0 | 0.1217 |
| first event per player (independent observations) | injury_category (v1, original keywords) | 2.6646 | 3 | 2061 | 2061 | 0.4463 | -0.0002 |
| first event per player (independent observations) | position_group | 17.3425 | 4 | 2061 | 2061 | 0.0017 | 0.0065 |

## Supplementary Table S5. Dunn pairwise comparisons: injury category

| **Group A** | **Group B** | **n A** | **n B** | **Players A** | **Players B** | **z** | **p (unadjusted)** | **p (Bonferroni, m = 6)** |
| --- | --- | --- | --- | --- | --- | --- | --- | --- |
| Muscle/tendon | Joint/ligament | 4698 | 2594 | 1463 | 1295 | -4.0513 | 0.0001 | 0.0003 |
| Muscle/tendon | Illness | 4698 | 1732 | 1463 | 1023 | -3.7873 | 0.0002 | 0.0009 |
| Muscle/tendon | Other | 4698 | 3924 | 1463 | 1515 | -5.1733 | 0.0 | 0.0 |
| Joint/ligament | Illness | 2594 | 1732 | 1295 | 1023 | -0.2373 | 0.8125 | 1.0 |
| Joint/ligament | Other | 2594 | 3924 | 1295 | 1515 | -0.505 | 0.6136 | 1.0 |
| Illness | Other | 1732 | 3924 | 1023 | 1515 | -0.1878 | 0.8511 | 1.0 |

Tests treat episodes as independent (exploratory).

## Supplementary Table S6. Dunn pairwise comparisons: injury-duration group

| **Group A** | **Group B** | **n A** | **n B** | **Players A** | **Players B** | **z** | **p (unadjusted)** | **p (Bonferroni, m = 6)** |
| --- | --- | --- | --- | --- | --- | --- | --- | --- |
| 1-14 | 15-30 | 5795 | 3387 | 1629 | 1417 | 3.2069 | 0.0013 | 0.0081 |
| 1-14 | 31-90 | 5795 | 2832 | 1629 | 1402 | 3.7276 | 0.0002 | 0.0012 |
| 1-14 | >90 | 5795 | 934 | 1629 | 690 | 5.209 | 0.0 | 0.0 |
| 15-30 | 31-90 | 3387 | 2832 | 1417 | 1402 | 0.6324 | 0.5271 | 1.0 |
| 15-30 | >90 | 3387 | 934 | 1417 | 690 | 3.0928 | 0.002 | 0.0119 |
| 31-90 | >90 | 2832 | 934 | 1402 | 690 | 2.6025 | 0.0093 | 0.0555 |

Tests treat episodes as independent (exploratory).

## Supplementary Table S7. Dunn pairwise comparisons: age group

| **Group A** | **Group B** | **n A** | **n B** | **Players A** | **Players B** | **z** | **p (unadjusted)** | **p (Bonferroni, m = 6)** |
| --- | --- | --- | --- | --- | --- | --- | --- | --- |
| <23 | 23-27 | 4407 | 5835 | 1421 | 1389 | 30.1664 | 0.0 | 0.0 |
| <23 | 28-30 | 4407 | 1839 | 1421 | 559 | 43.2061 | 0.0 | 0.0 |
| <23 | >30 | 4407 | 855 | 1421 | 231 | 39.7752 | 0.0 | 0.0 |
| 23-27 | 28-30 | 5835 | 1839 | 1389 | 559 | 22.3396 | 0.0 | 0.0 |
| 23-27 | >30 | 5835 | 855 | 1389 | 231 | 24.1499 | 0.0 | 0.0 |
| 28-30 | >30 | 1839 | 855 | 559 | 231 | 6.9321 | 0.0 | 0.0 |

Tests treat episodes as independent (exploratory).

## Supplementary Table S8. Random-intercept (player) mixed model with Model 4 covariates

| **Variable** | **Level** | **B** | **CI low** | **CI high** | **p** |
| --- | --- | --- | --- | --- | --- |
| Intercept |  | 135.618 | 116.31 | 154.926 | 0.0 |
| duration_group | 15-30 | -6.696 | -14.212 | 0.819 | 0.081 |
| duration_group | 31-90 | -17.758 | -26.019 | -9.498 | 0.0 |
| duration_group | >90 | -21.897 | -34.624 | -9.17 | 0.001 |
| injury_category | Illness | -0.832 | -11.012 | 9.347 | 0.873 |
| injury_category | Joint/ligament | -7.364 | -15.973 | 1.246 | 0.094 |
| injury_category | Other | -6.908 | -14.506 | 0.689 | 0.075 |
| age_c |  | -20.583 | -22.089 | -19.076 | 0.0 |
| baseline_m |  | -3.525 | -3.8 | -3.249 | 0.0 |
| Group Var |  | 0.392 | 0.338 | 0.447 | 0.0 |

Converged: True; player variance 10361; residual variance 26404; ICC = 0.282 (REML).

## Supplementary Table S9. Sensitivity analyses: key coefficients (B [95% CI]) of the Model 4 specification

| **Analysis** | **n** | **R²** | **age_c** | **baseline_m** | **duration_30** | **duration_group: 15-30** | **duration_group: 31-90** | **duration_group: >90** | **injury_category: Illness** | **injury_category: Joint Injury** | **injury_category: Joint/ligament** | **injury_category: Other** |
| --- | --- | --- | --- | --- | --- | --- | --- | --- | --- | --- | --- | --- |
| S0 main M4 | 12,936 | 0.177 | -18.24 [-19.67, -16.80] | -1.94 [-2.30, -1.58] | — | -11.41 [-19.59, -3.23] | -23.59 [-32.42, -14.76] | -26.36 [-42.59, -10.13] | 0.98 [-10.35, 12.32] | — | -5.44 [-14.36, 3.47] | -8.86 [-17.06, -0.66] |
| S1 first event per player | 2,059 | 0.160 | -34.52 [-39.16, -29.89] | -4.94 [-6.54, -3.34] | — | -11.67 [-49.68, 26.33] | -76.15 [-112.57, -39.73] | -54.71 [-108.13, -1.30] | -0.66 [-54.32, 53.00] | — | -6.52 [-42.00, 28.97] | -22.40 [-57.45, 12.66] |
| S10 age as groups | 12,936 | 0.167 | — | -1.81 [-2.15, -1.48] | — | -11.33 [-19.55, -3.11] | -22.35 [-31.26, -13.44] | -26.07 [-42.30, -9.84] | 1.49 [-9.81, 12.78] | — | -3.46 [-12.40, 5.49] | -6.32 [-14.57, 1.93] |
| S11 age B-spline df=4 | 12,936 | 0.199 | — | -1.73 [-2.06, -1.40] | — | -10.58 [-18.68, -2.48] | -23.76 [-32.43, -15.09] | -23.93 [-39.95, -7.92] | 0.11 [-11.12, 11.34] | — | -4.81 [-13.63, 4.02] | -8.35 [-16.41, -0.30] |
| S12 clinical diagnoses only | 11,568 | 0.182 | -18.58 [-20.09, -17.07] | -1.95 [-2.31, -1.59] | — | -11.55 [-20.45, -2.65] | -24.58 [-34.09, -15.07] | -29.79 [-46.30, -13.28] | 0.26 [-11.15, 11.67] | — | -6.01 [-14.97, 2.95] | -1.38 [-11.03, 8.27] |
| S13 untrimmed, winsorised at trim bounds | 13,191 | 0.170 | -24.65 [-26.84, -22.46] | -2.62 [-3.12, -2.11] | — | -13.55 [-24.35, -2.76] | -28.35 [-40.42, -16.28] | -45.95 [-65.02, -26.89] | 0.24 [-15.54, 16.02] | — | -6.31 [-19.24, 6.62] | -17.19 [-27.70, -6.67] |
| S15a M1 first event | 2,059 | 0.093 | -35.15 [-39.30, -31.00] | — | -10.82 [-18.51, -3.12] | — | — | — | 19.76 [-33.41, 72.94] | — | -0.16 [-36.41, 36.10] | -9.69 [-44.25, 24.87] |
| S15b M2 first event | 2,059 | 0.098 | -35.37 [-39.69, -31.06] | — | -10.75 [-18.55, -2.95] | — | — | — | 18.70 [-34.44, 71.83] | — | -0.42 [-36.81, 35.97] | -11.18 [-46.20, 23.84] |
| S15c M3 first event | 2,059 | 0.121 | -30.39 [-34.49, -26.30] | -4.70 [-5.89, -3.51] | -13.29 [-20.98, -5.60] | — | — | — | 6.38 [-45.97, 58.73] | — | -9.10 [-44.96, 26.77] | -22.22 [-56.35, 11.92] |
| S2 untrimmed sample | 13,191 | 0.053 | -37.32 [-45.63, -29.01] | -3.90 [-4.99, -2.80] | — | -20.56 [-45.15, 4.04] | -37.66 [-74.27, -1.04] | -49.41 [-103.90, 5.09] | 8.78 [-47.35, 64.91] | — | -1.66 [-39.44, 36.13] | -35.35 [-59.54, -11.16] |
| S3 outcome 100*log(post/pre) | 12,936 | 0.309 | -8.64 [-9.12, -8.16] | -0.78 [-0.92, -0.64] | — | -6.17 [-8.72, -3.62] | -12.07 [-14.90, -9.23] | -22.42 [-27.17, -17.66] | -2.14 [-5.69, 1.40] | — | -1.75 [-4.82, 1.32] | -3.43 [-6.13, -0.73] |
| S4 log(post) ~ log(pre) + covariates | 12,936 | 0.861 | -0.08 [-0.08, -0.08] | — | — | -0.08 [-0.10, -0.06] | -0.15 [-0.17, -0.12] | -0.26 [-0.30, -0.21] | -0.05 [-0.09, -0.02] | — | -0.03 [-0.06, -0.00] | -0.06 [-0.09, -0.04] |
| S5 original age variable (valuation-record age) | 12,936 | 0.176 | -18.13 [-19.55, -16.71] | -1.94 [-2.31, -1.58] | — | -11.41 [-19.58, -3.23] | -23.40 [-32.24, -14.57] | -26.22 [-42.47, -9.98] | 1.54 [-9.81, 12.88] | — | -5.70 [-14.62, 3.21] | -8.95 [-17.16, -0.74] |
| S6 original keyword mapping v1 | 12,936 | 0.177 | -18.23 [-19.67, -16.80] | -1.94 [-2.30, -1.58] | — | -11.25 [-19.43, -3.07] | -23.16 [-31.97, -14.35] | -25.64 [-41.98, -9.31] | 3.90 [-8.49, 16.29] | -5.64 [-14.65, 3.36] | — | -8.76 [-16.82, -0.70] |
| S7 one event per pre/post valuation pair | 11,223 | 0.178 | -18.83 [-20.28, -17.38] | -2.06 [-2.43, -1.68] | — | -11.59 [-20.73, -2.45] | -24.69 [-34.07, -15.32] | -26.94 [-42.72, -11.17] | 4.93 [-7.98, 17.83] | — | -4.55 [-14.12, 5.02] | -9.78 [-18.82, -0.75] |
| S8 start-anchored window (pre in [start-120,start), post in (start,start+120]) | 7,389 | 0.136 | -3.99 [-4.43, -3.56] | -0.44 [-0.53, -0.35] | — | -4.73 [-7.62, -1.83] | -10.17 [-13.01, -7.33] | -15.47 [-19.73, -11.20] | -2.70 [-6.00, 0.59] | — | -0.22 [-3.31, 2.86] | -2.50 [-5.32, 0.32] |
| S8b main outcome on the S8 subsample | 7,389 | 0.210 | -22.13 [-24.09, -20.17] | -2.47 [-2.94, -1.99] | — | -15.97 [-27.94, -4.01] | -35.88 [-48.75, -23.02] | -36.94 [-60.49, -13.38] | 0.83 [-15.82, 17.49] | — | -11.44 [-24.39, 1.50] | -16.31 [-28.05, -4.57] |
| S9 rounded original outcome | 12,936 | 0.177 | -18.24 [-19.67, -16.80] | -1.94 [-2.30, -1.58] | — | -11.42 [-19.59, -3.24] | -23.58 [-32.41, -14.75] | -26.35 [-42.58, -10.12] | 0.98 [-10.36, 12.31] | — | -5.44 [-14.36, 3.47] | -8.86 [-17.06, -0.66] |

S3 outcome is 100·log(post/pre); S4 outcome is log(post) with log(pre) as covariate; S8 uses the start-anchored ±120-day window.

## Supplementary Table S10. Matched-control difference-in-differences (main matching specification)

| **Estimate** | **Level** | **B (log)** | **CI low** | **CI high** | **p** | **% equivalent** | **% CI low** | **% CI high** | **Matched episodes** |
| --- | --- | --- | --- | --- | --- | --- | --- | --- | --- |
| DiD log(post/pre): injured minus control | — | -0.0447 | -0.0696 | -0.0198 | 0.0004 | -4.3727 | -6.727 | -1.9591 | 6689.0 |
| injured: mean log change | — | 0.2315 | 0.2072 | 0.2558 | 0.0 | 26.0492 | 23.0279 | 29.1447 | 6689.0 |
| control: mean log change | — | 0.2762 | 0.253 | 0.2994 | 0.0 | 31.813 | 28.787 | 34.9102 | 6689.0 |
| DiD percentage points: injured minus control | — | -22.1145 | -35.565 | -8.664 | 0.0013 | — | — | — | 6689.0 |
| pre-trend difference (log, 360 d before pre-valuation) | — | -0.0181 | -0.0508 | 0.0147 | 0.2791 | -1.7889 | -4.9487 | 1.476 | 6116.0 |
| DiD log by injury_category | Illness | -0.0304 | -0.0857 | 0.0248 | 0.2804 | -2.9955 | -8.2092 | 2.5142 | 6689.0 |
| DiD log by injury_category | Joint/ligament | -0.0744 | -0.1214 | -0.0275 | 0.0019 | -7.1745 | -11.4322 | -2.7122 | 6689.0 |
| DiD log by injury_category | Muscle/tendon | -0.0221 | -0.0579 | 0.0136 | 0.2253 | -2.1876 | -5.6244 | 1.3743 | 6689.0 |
| DiD log by injury_category | Other | -0.0578 | -0.0986 | -0.0169 | 0.0056 | -5.6119 | -9.3879 | -1.6785 | 6689.0 |
| heterogeneity test: DiD log across injury_category | — | 1.4923 | — | — | 0.2148 | — | — | — | — |
| DiD log by duration_group | 1-14 | 0.0213 | -0.0117 | 0.0542 | 0.2057 | 2.1488 | -1.1611 | 5.5696 | 6689.0 |
| DiD log by duration_group | 15-30 | -0.0585 | -0.1025 | -0.0145 | 0.0092 | -5.6837 | -9.7446 | -1.4401 | 6689.0 |
| DiD log by duration_group | 31-90 | -0.0777 | -0.1201 | -0.0353 | 0.0003 | -7.4791 | -11.3195 | -3.4724 | 6689.0 |
| DiD log by duration_group | >90 | -0.3139 | -0.3986 | -0.2292 | 0.0 | -26.9412 | -32.8738 | -20.4843 | 6689.0 |
| heterogeneity test: DiD log across duration_group | — | 19.8761 | — | — | 0.0 | — | — | — | — |
| DiD log by age_group | 23-27 | -0.0619 | -0.0944 | -0.0294 | 0.0002 | -6.0032 | -9.0071 | -2.9001 | 6689.0 |
| DiD log by age_group | 28-30 | -0.0671 | -0.121 | -0.0131 | 0.0149 | -6.4882 | -11.4007 | -1.3034 | 6689.0 |
| DiD log by age_group | <23 | -0.0223 | -0.0724 | 0.0278 | 0.3827 | -2.2064 | -6.9869 | 2.8198 | 6689.0 |
| DiD log by age_group | >30 | 0.0579 | -0.0265 | 0.1423 | 0.1785 | 5.9613 | -2.6119 | 15.2891 | 6689.0 |
| heterogeneity test: DiD log across age_group | — | 2.6696 | — | — | 0.0462 | — | — | — | — |
| adjusted DiD model (log) | — | 0.2219 | -0.103 | 0.5469 | 0.1806 | 24.85 | -9.7905 | 72.7926 | 6689.0 |
| adjusted DiD model (log) | 15-30 | -0.0955 | -0.1457 | -0.0452 | 0.0002 | -9.1069 | -13.5616 | -4.4226 | 6689.0 |
| adjusted DiD model (log) | 31-90 | -0.1186 | -0.1723 | -0.0649 | 0.0 | -11.1861 | -15.8294 | -6.2866 | 6689.0 |
| adjusted DiD model (log) | >90 | -0.356 | -0.4486 | -0.2634 | 0.0 | -29.95 | -36.1459 | -23.1529 | 6689.0 |
| adjusted DiD model (log) | Illness | -0.0722 | -0.1385 | -0.0059 | 0.0329 | -6.9642 | -12.9341 | -0.585 | 6689.0 |
| adjusted DiD model (log) | Joint/ligament | -0.0241 | -0.0794 | 0.0312 | 0.3935 | -2.3779 | -7.6297 | 3.1725 | 6689.0 |
| adjusted DiD model (log) | Other | -0.0555 | -0.1064 | -0.0047 | 0.0323 | -5.4035 | -10.0931 | -0.4693 | 6689.0 |
| adjusted DiD model (log) | — | -0.0036 | -0.0109 | 0.0036 | 0.3269 | -0.3623 | -1.0831 | 0.3637 | 6689.0 |
| adjusted DiD model (log) | — | -0.0106 | -0.0306 | 0.0094 | 0.2997 | -1.0529 | -3.0135 | 0.9473 | 6689.0 |

## Supplementary Table S10b. Matching specifications

| **matching_spec** | **matched_events** | **matched_share** | **injured_players** | **distinct_controls** | **max_control_reuse** | **DiD_log** | **CI_low** | **CI_high** | **p** | **pct_equivalent** | **pretrend_diff_log** | **pretrend_CI_low** | **pretrend_CI_high** | **unmatched_no_control** | **unmatched_injured_pre_lag** |
| --- | --- | --- | --- | --- | --- | --- | --- | --- | --- | --- | --- | --- | --- | --- | --- |
| main: league exact, age +-1, caliper 0.20 | 6689 | 0.5166 | 1732 | 1671 | 25 | -0.0447 | -0.0696 | -0.0198 | 0.0004 | -4.3727 | -0.0181 | -0.0508 | 0.0147 | 6133 | 126 |
| S-a: caliper 0.50 | 9821 | 0.7585 | 1927 | 1855 | 39 | -0.0606 | -0.0815 | -0.0398 | 0.0 | -5.8812 | -0.0213 | -0.0493 | 0.0068 | 3001 | 126 |
| S-b: any league | 11379 | 0.8788 | 1999 | 1969 | 46 | -0.0639 | -0.0837 | -0.044 | 0.0 | -6.1884 | -0.0022 | -0.027 | 0.0226 | 1443 | 126 |
| S-c: any league, age +-2, caliper 0.50 | 12536 | 0.9682 | 2039 | 1996 | 46 | -0.0674 | -0.0862 | -0.0486 | 0.0 | -6.5139 | -0.0063 | -0.0295 | 0.017 | 286 | 126 |

## Supplementary Table S11. Balance after matching

| **variable** | **injured_mean** | **control_mean** | **SMD_post_match** | **note** |
| --- | --- | --- | --- | --- |
| age at index date (years) | 23.946 | 23.969 | -0.007 |  |
| log baseline value | 15.403 | 15.407 | -0.003 |  |
| pre-valuation lag from injury start (days) | 198.066 | 198.293 | -0.004 |  |
| post-valuation lag from injury end (days) | 202.706 | 202.822 | -0.002 |  |
| league / position group |  |  | 0.0 | exact match |
| control reuse: distinct controls / max uses / median uses | 1671.0 | 25.0 | 3.0 |  |

## Supplementary Table S12. Matched versus unmatched episodes and reasons for non-matching

| **matched** | **n** | **age_mean** | **baseline_median_m** | **duration_median** | **y_mean** | **y_median** | **share_under23** |
| --- | --- | --- | --- | --- | --- | --- | --- |
| 0.0 | 6259.0 | 24.95 | 8.0 | 16.0 | 55.96 | 0.0 | 0.32 |
| 1.0 | 6689.0 | 23.95 | 6.0 | 16.0 | 72.96 | 10.0 | 0.36 |
| **reason** | | | | **n_events** | | | |
| no control within caliper | | | | 6133 | | | |
| injured pre-valuation older than 365 days | | | | 114 | | | |
| no stratum / missing age | | | | 12 | | | |

## Supplementary Table S13. Timing of pre- and post-injury valuations relative to the injury (days)

| **group** | **measure** | **n** | **mean** | **sd** | **median** | **q1** | **q3** | **min** | **max** |
| --- | --- | --- | --- | --- | --- | --- | --- | --- | --- |
| All events | pre_lag_days | 12948 | 201.2 | 63.7 | 191.0 | 152.0 | 240.0 | 120.0 | 1787.0 |
| All events | post_lag_from_start_days | 12919 | 233.1 | 70.7 | 226.0 | 181.0 | 272.0 | -180.0 | 1399.0 |
| All events | post_lag_from_end_days | 12919 | 203.0 | 56.7 | 195.0 | 157.0 | 242.0 | 121.0 | 1233.0 |
| All events | valuation_interval_days | 12919 | 434.3 | 100.3 | 416.0 | 363.0 | 504.0 | 0.0 | 2198.0 |
| duration 1-14 | pre_lag_days | 5795 | 199.7 | 59.6 | 191.0 | 151.0 | 238.0 | 120.0 | 948.0 |
| duration 1-14 | post_lag_from_start_days | 5791 | 209.8 | 55.8 | 204.0 | 163.0 | 249.0 | 121.0 | 940.0 |
| duration 1-14 | post_lag_from_end_days | 5791 | 203.0 | 55.7 | 197.0 | 156.0 | 243.0 | 121.0 | 928.0 |
| duration 1-14 | valuation_interval_days | 5791 | 409.5 | 85.5 | 383.0 | 356.0 | 464.0 | 247.0 | 1368.0 |
| duration 15-30 | pre_lag_days | 3387 | 201.2 | 60.3 | 192.0 | 153.0 | 240.0 | 120.0 | 1136.0 |
| duration 15-30 | post_lag_from_start_days | 3385 | 224.9 | 56.0 | 219.0 | 179.0 | 265.0 | 135.0 | 594.0 |
| duration 15-30 | post_lag_from_end_days | 3385 | 205.0 | 55.8 | 198.0 | 158.0 | 245.0 | 121.0 | 566.0 |
| duration 15-30 | valuation_interval_days | 3385 | 426.1 | 87.8 | 411.0 | 360.0 | 490.0 | 259.0 | 1328.0 |
| duration 31-90 | pre_lag_days | 2832 | 202.4 | 71.6 | 190.0 | 152.0 | 242.0 | 120.0 | 1787.0 |
| duration 31-90 | post_lag_from_start_days | 2830 | 251.5 | 57.3 | 245.0 | 210.0 | 289.0 | 108.0 | 867.0 |
| duration 31-90 | post_lag_from_end_days | 2830 | 201.9 | 55.2 | 193.0 | 160.0 | 239.0 | 121.0 | 814.0 |
| duration 31-90 | valuation_interval_days | 2830 | 453.9 | 97.2 | 443.0 | 373.0 | 526.0 | 250.0 | 1989.0 |
| duration >90 | pre_lag_days | 934 | 206.6 | 73.7 | 192.5 | 156.0 | 246.8 | 120.0 | 1201.0 |
| duration >90 | post_lag_from_start_days | 913 | 353.8 | 99.7 | 338.0 | 285.0 | 408.0 | -180.0 | 1399.0 |
| duration >90 | post_lag_from_end_days | 913 | 199.2 | 69.8 | 185.0 | 153.0 | 234.0 | 121.0 | 1233.0 |
| duration >90 | valuation_interval_days | 913 | 560.9 | 130.2 | 546.0 | 488.0 | 618.0 | 0.0 | 2198.0 |
| All events | events_sharing_same_pre_post_valuation_pair_within_player | 3202 |  |  |  |  |  |  |  |
| All events | distinct_pre_post_valuation_pairs | 11233 |  |  |  |  |  |  |  |
| All events | post_valuation_before_end_date (should be 0) | 0 |  |  |  |  |  |  |  |

## Supplementary Figures

Figure S1. Composition of the 'other' category (revised dictionary): median (bars) and mean (circles) market-value change by sub-group.

Figure S2. Model 4 coefficients with player-clustered 95% confidence intervals (position, season and league terms omitted).

Figure S3. Fifteen most frequent diagnoses: mean market-value change with player-cluster bootstrap 95% confidence intervals and medians.

## STROBE checklist (cohort studies)

| **Item** | **STROBE recommendation** | **Manuscript section** | **Where addressed in this revision** |
| --- | --- | --- | --- |
| 1a | Indicate the study design with a commonly used term in the title or abstract | Title; Abstract | Title revised to 'retrospective cohort study'; 'trajectories' replaced by 'change' |
| 1b | Informative and balanced summary in the abstract | Abstract | Abstract rewritten from result_summary.md (sample; window; clustered inference; DiD) |
| 2 | Scientific background and rationale | Introduction | Association framing; no causal claims |
| 3 | Specific objectives and prespecified hypotheses | Introduction (end) | Objectives: association of duration/category with change; interaction hypotheses stated as exploratory |
| 4 | Key elements of study design | Methods 2.1 | Retrospective cohort of injury episodes; matched-control DiD exploratory |
| 5 | Setting; locations; dates of recruitment; exposure; follow-up; data collection | Methods 2.2 | Transfermarkt scrape 2025-01-18; 2024 squads of five leagues; injuries 2008-2024 (cohort_definition.md) |
| 6a | Eligibility criteria; sources and methods of selection; follow-up | Methods 2.2-2.3 | Squad selection frame; SQL filter; trimming; exclusion_log.csv; cohort_flow.csv |
| 6b | Matching criteria and number of exposed/unexposed (cohort) | Methods 2.6 | League/position/age +-1/log-value caliper 0.20; 6689 matched (TableS_did_matching_specs) |
| 7 | Clearly define all outcomes; exposures; predictors; confounders; effect modifiers | Methods 2.3 | y_exact; duration groups; mapping v2; age exact; position; baseline; season; league group |
| 8 | Sources of data and details of methods of assessment | Methods 2.2-2.3 | Valuation-window rule verified (audit_report §4); age definition |
| 9 | Describe any efforts to address potential sources of bias | Methods 2.4 / 2.6 | Clustered SE; first-event sensitivity; matched controls; pre-trend check |
| 10 | Explain how the study size was arrived at | Methods 2.2 | All eligible episodes; no power calculation |
| 11 | Explain how quantitative variables were handled | Methods 2.3 | Duration and age groups (boundaries stated); centring; scaling |
| 12a | Describe all statistical methods including confounding control | Methods 2.4 | M1-M5 formulas; cluster covariance; Wald tests |
| 12b | Methods used to examine subgroups and interactions | Methods 2.4 | Joint Wald tests; standardised contrasts; Holm |
| 12c | How missing data were addressed | Methods 2.3 | Complete-case (12 episodes without DOB/position); missing league as 'Unknown' level |
| 12d | Cohort: how loss to follow-up was addressed | Methods 2.3 | Episodes without a post-window valuation excluded (1635); reported in flow |
| 12e | Sensitivity analyses | Methods 2.5 | S1-S15 (TableS_sensitivity_*) |
| 13a | Numbers of individuals at each stage | Results 3.1 | 15327 -> 13205 -> 12948 (cohort_flow) |
| 13b | Reasons for non-participation at each stage | Results 3.1 | exclusion_log reasons |
| 13c | Consider use of a flow diagram | Results 3.1 / Figure | Flow from cohort_flow.csv |
| 14a | Characteristics of study participants and information on exposures and confounders | Results 3.1 / Table 1 | Table1A-E with n events; n players; mean/SD; median/IQR |
| 14b | Number of participants with missing data for each variable | Results 3.1 | cohort_manifest.json missing_in_analysis |
| 14c | Cohort: summarise follow-up time | Results 3.1 | valuation_timing_lags.csv |
| 15 | Report numbers of outcome events or summary measures over time | Results 3.2 | Table 1; overall_summary |
| 16a | Unadjusted and adjusted estimates with precision (95% CI) | Results 3.4 / Table 2 | Table2_revised_models_journal; Table2_original_models_plain_vs_cluster_SE |
| 16b | Report category boundaries when continuous variables were categorised | Methods 2.3 | Duration 1-14/15-30/31-90/>90; age <23/23-27/28-30/>30 |
| 16c | Consider translating relative risk into absolute risk | Results | Percentage-point differences; DiD as % equivalent |
| 17 | Report other analyses done (subgroups; interactions; sensitivity) | Results 3.5-3.7 | Table 3; TableS_sensitivity; TableS_did |
| 18 | Summarise key results with reference to objectives | Discussion | Rewritten |
| 19 | Discuss limitations including bias direction and magnitude | Discussion (Limitations) | Revised limitation list (revision_notes.md §3) |
| 20 | Give a cautious overall interpretation | Discussion | Association-level wording; age-specific recommendation removed |
| 21 | Discuss generalisability | Discussion | Five-league 2024 squads; survivor selection |
| 22 | Give the source of funding and role of funders | Funding statement |  |
